# Stress peptides sensitize fear circuitry to promote passive coping

**DOI:** 10.1038/s41380-018-0089-2

**Published:** 2018-06-14

**Authors:** Pinelopi Pliota, Vincent Böhm, Florian Grössl, Johannes Griessner, Ornella Valenti, Klaus Kraitsy, Joanna Kaczanowska, Manuel Pasieka, Thomas Lendl, Jan M. Deussing, Wulf Haubensak

**Affiliations:** 1 Research Institute of Molecular Pathology (IMP), Vienna Biocenter (VBC), Dr. Bohr Gasse 7, 1030 Vienna, Austria; 2 Division of Neurophysiology and Neuropharmacology, Centre for Physiology and Pharmacology, Medical Univ. Vienna, Schwarzspanierstrasse 17, 1090 Vienna, Austria; 3 Preclinical Phenotyping, Vienna Biocenter (VBC), Dr. Bohr Gasse 3, 1030 Vienna, Austria; 4 Bioinformatics and Scientific Computing, Vienna Biocenter (VBC), Dr. Bohr Gasse 3, 1030 Vienna, Austria; 5 Department of Stress Neurobiology & Neurogenetics, Max Planck Institute of Psychiatry, 80804 Munich, Germany

**Keywords:** Neuroscience, Psychology

## Abstract

Survival relies on optimizing behavioral responses through experience. Animals often react to acute stress by switching to passive behavioral responses when coping with environmental challenge. Despite recent advances in dissecting mammalian circuitry for Pavlovian fear, the neuronal basis underlying this form of non-Pavlovian anxiety-related behavioral plasticity remains poorly understood. Here, we report that aversive experience recruits the posterior paraventricular thalamus (PVT) and corticotropin-releasing hormone (CRH) and sensitizes a Pavlovian fear circuit to promote passive responding. Site-specific lesions and optogenetic manipulations reveal that PVT-to-central amygdala (CE) projections activate anxiogenic neuronal populations in the CE that release local CRH in response to acute stress. CRH potentiates basolateral (BLA)-CE connectivity and antagonizes inhibitory gating of CE output, a mechanism linked to Pavlovian fear, to facilitate the switch from active to passive behavior. Thus, PVT-amygdala fear circuitry uses inhibitory gating in the CE as a shared dynamic motif, but relies on different cellular mechanisms (postsynaptic long-term potentiation vs. presynaptic facilitation), to multiplex active/passive response bias in Pavlovian and non-Pavlovian behavioral plasticity. These results establish a framework promoting stress-induced passive responding, which might contribute to passive emotional coping seen in human fear- and anxiety-related disorders.

## Introduction

Animals integrate past experiences to adapt behavioral responses to future environmental challenges. The type of responding depends on various factors, but can be classified broadly as either active or passive. In a threatening context, active response entails exploration, hyperactivity, and “fight-or-flight” responses, while passive responses reduce exploration and promote freezing [1, 2]. The choice between active and passive responding typically depends on previous experience [1].

Pavlovian experiences form an associative memory between a predictive cue and an aversive event, for instance between an auditory cue and inescapable electric foot shock. This discrete memory drives long-lasting cue directed fear and bias for passive freezing responses. Maladaptation of this process has been linked to posttraumatic stress disorder (PTSD) in humans [3]. The last decade yielded considerable progress in understanding neuronal circuit dynamics and molecular control underlying this form of behavioral plasticity. Functional neuroanatomy of Pavlovian fear, from tone/foot shock conditioning in animal models to human imaging experiments, has identified BLA-CE circuitry as central element for integrating fearful stimuli and controlling fear behaviors [4, 5]. Amygdala connectivity [6] and BLA−CE interactions [7] are strongly modulated by Pavlovian learning through postsynaptic processes in BLA [8] and CE [7, 9]. The CE, in turn, mediates fear [10, 11], and fear-related active/passive behavioral responses [9, 12, 13] through antagonistic inhibitory circuit elements (most prominently somatostatin (SOM)^+^, CRH^+^ and protein kinase Cδ (PKCδ)^+^ neurons in the lateral CE (CEl) and output neurons in the medial CE (CEm)). However, despite recent progress, we are only at the beginning of understanding how their interactions control the multitude of CE-dependent behaviors [7, 9, 11–15]. In the context of fear behavior, the overarching view is that CEl neurons (potentially PKCδ^+^ neurons [11]) gate amygdala function by antagonizing BLA-to-CEm signaling, which, in turn, drives freezing [5, 16].

**Fig. 1:**
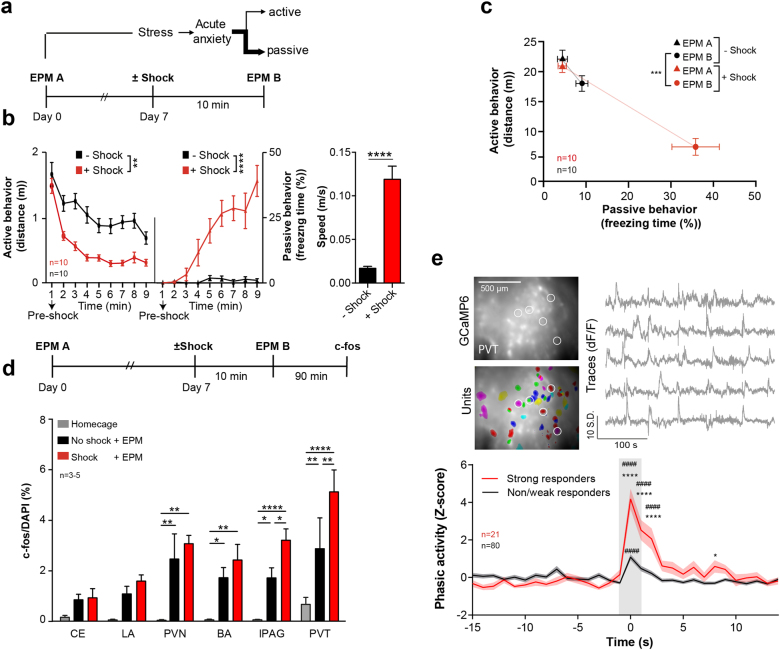
Shock experience modulates behavior and recruits forebrain stress circuits. **a**−**c** Stress modulation of behavior. **a** The behavioral paradigm for stress-induced behavioral adaptation. Top, previous experience affects the behavioral response to environmental challenges. This study focuses on stress-induced non-Pavlovian plasticity. Bottom, naïve mice were exposed to EPM (EPM A). A week later they were re-exposed to the same apparatus (EPM B) after receiving foot shocks in a different context. Control mice were exposed to the shock context but did not receive any foot shocks. **b** Immediate shift from active to passive behaviors during shock experience in the shock context. Left, note the decrease in active (left) and the increase in passive behaviors (right) across time in the shock group. During the first minute no shock was given to the mice; after that, shocks were pseudorandomized with a 20–100 s interval (unpaired *t* test_distance_ *p* = 0.0015, unpaired *t* test_freezing_ *p* < 0.0001). Right, immediate shock response given by the speed the mice displayed during the 1 s shocks (unpaired *t* test *p* < 0.0001). **c** Active (*y*-axis) and passive (x-axis) behavior during EPM A (triangles) and EPM B (circles). Shock-modulated active and passive behaviors in EPM B (red circle) (MANOVA *p* < 0.001). **d−e** Shock experience recruits PVT. **d** c-fos IEG screen 90 min after the exposure to the EPM B (top) identifies stress recruited limbic structures (bottom). RM two-way ANOVA *p*_treatment_ < 0.0001, Holm−Sidak post hoc test (*n* indicates averages from 3 to 5 animals). CE central nucleus of the amygdala, LA lateral amygdala, PVN paraventricular nucleus of the hypothalamus, BA basal amygdala, lPAG lateral periaqueductal gray, PVT paraventricular nucleus of the thalamus (bregma −1.3 to −1.7). **e** Deep brain calcium imaging of PVT in freely moving mice. Top, Deep brain images of PVT neurons expressing GCaMP6 (top, average projection), individual isolated units (bottom) and example traces (right) of isolated units (white circles in left panels). Note that in the average projections not all active units are visible. Bottom, mean population shock response. Phasic activity was calculated from Z-scores of events with the full recording session as baseline. Cells were classified as strong responders when Ca^2+^ events coincided with 15 s post shock intervals with >95 % confidence. All other cells were termed weak/non-responders (RM two-way ANOVA, *p*_interaction_ < 0.0001, Holm−Sidak post hoc test) (*n* indicates neurons extracted from five animals). # Comparison to first time points (at *t* = −15 s). The gray bar represents shock. Significance levels are given as */# *p* < 0.05, **/## *p* < 0.01, ***/### *p* < 0.001, ****/#### *p* < 0.0001

In the absence of predictive cues, exposure to a (then) unpredictable stressor evokes, instead of cue directed fear, generalized anxiety states (Fig. 1a). A hallmark of such experiences is that the organism adapts by shifting the strategy in coping with behavioral challenges. In rodents, unpredictable inescapable shocks typically promote passive responses. Such stress-induced behavioral adaptations occur in a variety of settings. Previous inescapable shock facilitates passive defensive responses (freezing) to innately aversive auditory stimuli [17] and alters coping strategies in avoidance paradigms [18]. In its most basic form, it switches behavioral responding in mazes [17, 19, 20]: after inescapable foot shock, animals avoid anxiogenic areas and reduce active behaviors, like exploration and rearing. In humans, this type of reaction is exacerbated in patients with generalized anxiety disorder (GAD)-related symptoms, who show a bias for passive emotional coping after stressful events [18, 21]. However, despite its relevance for human psychopathology, our understanding of the neuronal mechanisms underlying stress-induced anxiety-related passive responding, and their relation to Pavlovian fear processes, is still limited.

In a simplistic model, this form of behavioral plasticity may emerge from the interaction of brain stress pathways with centers controlling fear behaviors. The paraventricular thalamus (PVT) and the amygdala represent such an interconnected system [12, 22–24]. The PVT is a functionally and anatomically heterogenous structure [24], with anterior and posterior domains differentially controlling a variety of appetitive and aversive behaviors [25–29]. The PVT processes multimodal stress signals [27, 30–35] and modulates fear responses by direct projections to CE neurons [28, 36]. Moreover, afferent inputs from BLA to CE can directly control anxiety responses [37]. In addition, the CE is rich in various neuropeptides capable of modulating anxiety levels (e.g., enkephalin, neuropeptide Y and CRH) [38]. CRH acts as powerful modulator of intra-amygdala connectivity [39], facilitating glutamatergic inputs to CE neurons [40]. Likewise, CE CRH neurons play an important role in Pavlovian fear responses [13, 38]. Additionally, limbic CRH [41] and CRH receptor 1 (CRHR1) [42] have also long been identified as central regulators of anxiety. The central role for CRH in stress signaling (either related to fear or anxiety) highlights it as potential therapeutic target for stress-related diseases, including anxiety disorders [43]. Collectively, these data support a role for CRH and PVT/BLA-CE circuitry in controlling passive responding after an acute stressful experience.

To map brain circuitry mediating this behavioral adaptation, we performed an unbiased limbic system wide immediate early gene (IEG) screen. Subsequent genetic circuit dissection, in vivo Ca^2+^ imaging and ex vivo electrophysiology identified PVT-CE circuitry as central component processing shock signals. Investigating the modulation of local CE circuit dynamics by stress and neuropeptides indicated that stress and CRH sensitize BLA-CE circuitry. This antagonizes inhibitory gating of CE output by the CEl while facilitating synaptic transmission from BLA to CEm, which ultimately promotes passive responding. As these behavioral and circuit dynamic effects were partially reverted by CRHR1 antagonists, our work provides a mechanistic framework for CRHR1-directed therapeutics.

## Materials and methods

### Subjects

Male C57BL/6J or transgenic 2–4-month-old mice were used for all the experiments, unless otherwise indicated. Food and water were provided ad libitum. The animals were group-housed at 21 °C in a 14-h light/10-h dark cycle (light on at 0600 hours) with tests performed during the light cycle. C57BL/6J male mice were obtained post-weaning from the Institute of Molecular Pathology (IMP) mouse facility.

PKCδ::GluClα-CRE BAC transgenic mice (PKCδ::Cre) [11], Srt-ires-CRE (SOM::Cre) (Jackson Laboratory stock no: 013044), and CRH-ires-CRE line (CRH::Cre) (Jackson Laboratory stock no: 012704) were backcrossed to C57BL/6J background. To identify CRHR expression CRHR1-tau-lacZ mice were used [44]. All mice were heterozygous for the transgene. Region selective CRHR1 knock-out was performed by injecting AAV::Cre into homozygous Crhr1^loxP/loxP^ animals [44]. Cre-recombination in the target regions was reported by co-injecting Cre-dependent AAV::GFP.

All animal procedures were performed in accordance with institutional guidelines and were approved by the respective Austrian (BGBl nr. 501/1988, idF BGBl I no. 162/2005) and European (Directive 86/609/EEC of 24 November 1986, European Community) authorities and covered by the license M58/002220/2011/9.

### Behavioral manipulations

In preparation for behavioral experiments, all mice were previously handled by the experimenter. Experiments were performed on a custom-made elevated plus maze (EPM) (50 cm from ground, 30 cm arms). Light conditions were calibrated to 200 lux at the end of the open arm, and all tests performed in a sound-isolated room. Mice always started the test from the center field facing towards the open arms. Each assay lasted 12.5 min. After each trial, the apparatus was cleaned with water and 70% ethanol.

One week after the first EPM trial, mice were transferred to a different contextual environment where they received ten foot shocks, 0.5 mA, of 1 s at randomized intervals of 20−100 s with a total duration of 10 min (precision regulated animal shocker H13-15 (Coulbourn Instruments)). Shocks were pseudo-random and controlled by Arduino Uno interfaces and custom scripts. Mice were then transferred back to their home cage for 10 min (or as indicated otherwise) before they were re-exposed to the second EPM trial. Videos were recorded and all behavior was scored using the ANYmaze software (Stoelting Co). Behavior was scored automatically and classified as freezing when mice maintained a 98% whole body immobility for at least 2 s. Distance was measured as the distanced traveled by the center point of the mouse and open arm time was measured as time spend on the two open arms (middle zone was excluded).

Optogenetic experiments were performed on animals virally expressing ChR2 or Arch, with a 2.5 min laser OFF/ON duty cycle. ChR2 stimulation (473 nm or 447 nm laser) was performed with 20 ms pulses at a frequency of 10 Hz and a peak intensity of 10 mW at the end of the tip. Arch inactivation (598 nm laser) was performed with constant light at 5 mW. Behavior was analyzed for the On-periods (5 min total) and compared between experimental groups. Behavioral protocols, recording and lasers were controlled by Arduino boards running customized scripts coupled to the Anymaze (Stoelting) software for behavioral control and recording. Behavioral scoring was performed with Anymaze software. For better comparison of optogenetic experiments to other behavioral manipulations, distance measures during 2 × 2.5 min light On-phases were normalized to 12.5 min observation time by a factor of 2.5.

For pharmacogenetic manipulations, animals virally expressing hM3Dq DREADDs were injected with clozapine *N*-oxide CNO (5 μg/g mouse) 30 min prior behavioral experiments.

1–2 weeks prior experiments involving intracerebral drug administration, mice were bilaterally implanted with cannulas and habituated to the internal cannula (Bilaney Consultants GmbH, C313IS-5/Spc) insertion prior to the pump infusions (Harvard Apparatus Pump 11). The internal cannula insertion was performed in awake, but slightly restrained mice. For CE infusions, a volume of 30 nl a-helical CRH (Sigma Aldrich, concentration 15 mg/ml in aCSF) or CRH (Sigma Aldrich, concentration 100 mg/ml in aCSF) was injected at a rate of 15 nl/min. The controls went through the same manipulations but were infused only with aCSF.

### Immediate early gene circuit mapping

Ninety minutes after behavior, animals were perfusion fixed with 4% PFA in PBS. Serial forebrain cryosections were stained with anti-c-Fos primary (rabbit polyclonal, ab7963, Abcam, 1:1000) and anti-rabbit secondary antibodies (A-21202, Life Technologies or D3571, Invitrogen, 1:1000 + DAPI). For a more detailed description concerning transcardial perfusion, preparation of brain slices and immunohistochemistry see Supplementary experimental procedures.

Images were digitized using a Pannoramic 250 Flash (3D HISTECH Ltd, with 20/0.8 Plan-Apochromat objective) and analyzed using a semi-automated approach with custom scripts in a Definiens Developer XD environment (Definiens Software Suite). First, the nuclei were segmented on the DAPI channel using a LoG-Filter and watershed algorithms. On a set of training images, nuclei objects were manually classified as positive and negative and used as samples for training a machine-learning algorithm. Mean intensity, standard deviation, and local contrast parameters were extracted from it as input for a decision tree-based classifier. This classifier was then applied to all nuclei objects on a larger set of images to define positive cells. A similar approach was used to segment and classify cytoplasmic stain around the nuclei. Training was done separately for each channel. The output was given as the number of positive cells, either nuclear or cytoplasmic, co-staining of cells, total cell number for each image. Results of the automated cell counting were first analyzed using Graph Pad Prism^®^ (Version 6) to identify the number of positively stained nuclei for each mouse and region.

### Calcium imaging

Deep brain calcium imaging was performed with an nVistaHD 2.0 in vivo Rodent Brain Imaging System (Inscopix, Palo Alto, USA). GLP-0540 and GLP-0561 microendoscopic fibers were implanted for imaging PVT and CE, respectively.

CEl SOM^+^ and PKCδ^+^ neurons were imaged in SOM::Cre and PKCδ::Cre animals injected with Cre-dependent AAV for the expression of GCaMP6 (AAV1.Syn.Flex.GCaMP6f.WPRE.SV40). This approach allows reliable cell type-specific imaging of these cells (Supplementary Fig. 11a) [15]. CEm neurons were imaged in wt animals, injected with AAV for neuron-specific expression of GCaMP6 (AAV9.hsyn.GCaMP6m.WPRE) (Supplementary Fig. 11b). The absence of a genetic targeting strategy specific for CEm resulted in some remaining spatial ambiguity in discriminating CEm from CEl neurons. To reflect this, we refer to these cells as putative CEm neurons from here on.

After 1 week, the baseplate (BPL-2) was attached to the skull with dental cement. For habituation purposes before the day of the actual experiment, a dummy microscope was mounted to the implanted baseplate for 1 h. On the experimental day, the microscope was attached to the baseplate and the mouse proceeded with behavioral experiments. Behavioral control, Ca^2+^ and behavioral video recordings were performed on a fully synchronized custom built setup, running on Anymaze, Arduino 2.0 scripts and nVistaHD v2.0.32 software, respectively. Data were acquired at 15−24 fps. Movement artifacts were compensated by framewise image registration using a script in ImageJ macro language. During the first run, a reference image was created by aligning the first hundred frames and performing a Maximum Intensity Projection. This image was then used for aligning the whole timeseries. Features were enhanced applying a Laplacian of Gaussian filter, and the detection of Landmarks was performed via the “Extract Block Matching Correspondences” plugin in Fiji. The resulting transformations were then applied to the original images. In cases where no matching of the reference image with a frame could be achieved, the position of the original frame was used. The resulted video was further analyzed offline by employing the Mosaic analysis suite v1.1.3-1.2.0 (Inscopix, Palo Alto, USA), first by applying a Δ*F*/*F*_0_ normalization, where *F*_0_ was based on the entire length of the movie. A principal component analysis (PCA/ICA) separated images from individual cells. Event detection was performed on the isolated traces after cells were manually sorted. Ca^2+^ events were detected as Ca^2+^ signals with SD_local peak height_ > 3 and *τ*_decay_ > 1.5 s and served as proxy for neuronal activity. All further analysis was done in Neuroexplorer software (Plexon Inc.).

Tonic activity (Supplementary Fig. S2b, Supplementary Fig. S12a) represents the population activity of all cells in PVT, CEl, or CEm. Phasic activity (Figs. 1e, 5c, and Supplementary Fig. S2d, Supplementary Fig. 12b) was calculated from Z-scores of events with the full recording session as base. PVT cells were classified by analyzing peri-shock histograms and classified as strong responders when Ca^2+^ events coincided with 15 s post shock intervals with >95 % confidence, averaged across all shock trials/freezing incidents. If cells did not meet this criterion, they were termed weak/non-responders (Fig. 1e).

### Statistics

Samples sizes were chosen based on typical values for behavioral, histological, electrophysiological, or imaging analyses. All animals and samples were randomly assigned to experimental groups wherever possible. The experimenter was not blinded to the experimental group of the animals when handling. Behavioral, histological, electrophysiological, and imaging data were analyzed computationally unless indicated otherwise.

All statistics were performed in Graph Pad Prism (Version 6), Neuroexplorer or custom written R-codes. All statistical tests are indicated in the figure legends. Data were tested for normality using Kolmogorov−Smirnov at a significance level of *α* = 0.05, wherever possible, and given as mean ± SEM. For all parametric tests, the variances were assumed to be equal. If normality test did not pass, Kruskal−Wallis with Dunn’s post hoc tests was used, and data were given as median, interquartile ranges and 10−90 percentiles. Behavioral experiments were assessed by multivariate ANOVA with Hotteling’s T^2^ post hoc tests. Z-scores were assumed to be normal (reflecting population means) and compared by repeated measures two-way ANOVA with Holm−Sidak post hoc tests. If no significance is indicated, the test did not reach significance level of <0.05. Animals with incorrect lesion (cf. Supplementary Fig. S3), viral expression, wrong fiber placement (cf. Supplementary Fig. S4, S6, S10, S11a,b,c), or malfunctioning implants/probes/equipment were excluded from the analysis.

A detailed description for surgery, in vitro and in vivo electrophysiology and microdialysis is provided in supplementary experimental procedures.

## Results

We first sought to implement a simple and robust assay to investigate stress modulation of anxiety-related active passive response bias. A combination of inescapable foot shock stress experience followed by EPM challenge allows to monitor anxiety as well as active versus passive response bias from movement and freezing parameters [19, 20, 45]. Stress-induced anxiety and shift in active/passive responding was monitored when tracking animals’ behavior in sequential EPM challenges (EPM A and B), separated by exposure to ten repeated and unpredictable electrical foot shocks delivered in a novel shock context (Fig. 1a). During inescapable foot shocks, animals gradually exhibited more passive behaviors, with decreased exploration and increased freezing responses, which were absent in controls exposed to the same context but without shock (Fig. 1b). We next compared behaviors during exposure to pre-shock and post-shock EPM. While mice explored their environment actively in EPM A (Fig. 1c), they avoided open arms (Supplementary Fig. S1c) and behaved more passively in EPM B (Fig. 1c), exhibiting behavioral patterns they had previously adopted in the shock context (i.e., less active and more freezing, Fig. 1b). These data suggest that shock exposure leads to an anxiety-related shift to passive behaviors. Notably, shock-induced bias for passive behaviors (Fig. 1c) was transferred to subsequent EPM challenge, but was absent during resting time in home cage (Supplementary Fig. S1b). Thus, this bias cannot be explained by simple prolonged freezing state induced by the shock-exposure. Rather, it seems to reflect a passive responding towards behavioral EPM challenge after aversive experience and elevated anxiety. This effect persisted for at least 24 h but was strongest 10 min after the shock (Supplementary Fig. S1d).

We next sought to identify the most prominent limbic system regions involved in this stress-induced behavioral switch by an unbiased immediate early gene (c-fos) screen for areas activated by EPM (corresponding to EPM A) or shock and EPM (corresponding to EPM B). While the EPM or shock alone recruited most regions, previous shock experiences selectively potentiated activity within the lateral periaqueductal gray (lPAG) and the PVT (Fig. 1d), indicating that the lPAG and PVT were recruited during both stress and behavioral challenge. These results, and considering that the PVT is widely connected to other behavior-relevant nuclei [27] and functionally linked to stress responses [33–35], point towards a central role in mediating the stress-induced behavioral adaptations in our assay. Taking into account the functional organization of the PVT [25–29], we targeted our subsequent experiments to its posterior domain, which directly controls amygdala fear expression [26].

**Fig. 2:**
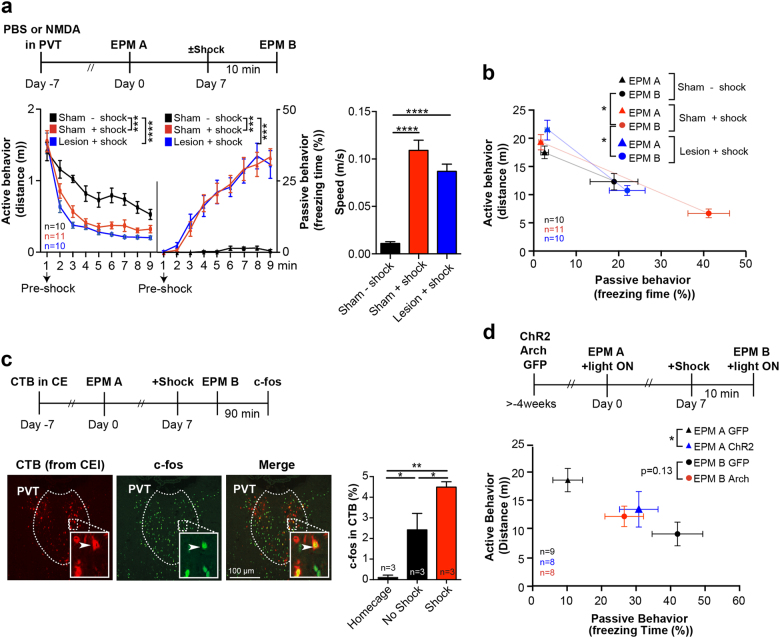
PVT-CE circuitry mediates stress-induced behavioral adaptation. **a**,**b** PVT is required for behavioral modulation after shock exposure. **a** Top, experimental timeline of the PVT lesioning experiment*.* Bottom left, in the shock-context, NMDA-lesions shock group**’**s active (left) and passive (right) behavior was indistinguishable from the sham-lesioned shock group but differed from the sham-lesioned no shock group (RM one-way ANOVA_freezing_ *p* < 0.0001 with Holm−Sidak, RM one-way ANOVA_freezing_ *p* = 0.0002 with Holm−Sidak). Bottom right, immediate shock response given by the speed the mice displayed during the 1 s shocks in the shock context (one-way ANOVA *p* < 0.0001, Holm−Sidak post hoc test). **b** Interaction of PVT lesion with delayed behavioral effects of shock experience. During behavioral challenge (EPM B) PVT lesioned group did not switch to passive behaviors compared to the sham-lesion shock group (MANOVA *p* < 0.0001, Hotelling**’**s T^2^ post hoc test). **c**, **d** The PVT-to-CEl projection modulates the delayed effect of stress experience. **c** Top, Experimental design for c-fos/CTB projection-specific activity mapping. Bottom left, c-fos/CTB labeled cells 90 min after exposure to the EPM B. Bottom right, recruitment of PVT-to-CEl projections by shock and behavioral challenge by projection-specific c-fos/CTB activity mapping (one-way ANOVA *p* = 0.0011, Holm−Sidak post hoc test). **d** Optogenetic manipulation of PVT-to-CEl projections in EPM A and EPM B. Top, ChR2, Arch or GFP virus injections with fiber implantations above CEl preceded the behavioral exposures. Light activation occurred in ON/OFF phases during both EPMs. Only the light ON phases were analyzed. (MANOVA *p* = 0.031, Hotelling**’**s T^2^ post hoc tests). Significance levels are given as *p < 0.05, **p < 0.01, ***p < 0.001, ****p < 0.0001

We next performed deep brain calcium imaging in freely moving mice to characterize PVT neuronal dynamics during shock and behavioral challenges. Ca^2+^ dynamics were recorded with a head-mounted miniature microendoscope in subjects expressing GCaMP6m in the PVT (Fig. 1e, Supplementary Fig. S2a). Neuronal Ca^2+^ events derived from individual traces revealed a subset of cells with phasic responses to shock (Fig. 1e, Supplementary Fig. S2c). We next examined neural traces of previous shock experiences during behavioral challenge by comparing calcium responses in freely moving animals during EPM A and B. PVT was more active during EPM B, the behavioral challenge that followed shock exposure (Supplementary Figure S2b). These results suggest that PVT relays stress signals for subsequent behavioral adaptations. Studies with NMDA lesioned animals support this view (Supplementary Fig. S3, Fig. 2a, b). While NMDA lesioned animals were indistinguishable from sham-controls during EPM A (Fig. 2b) and shock context exposure (Fig. 2a), they did not shift to passive behaviors in EPM B (Fig. 2b). These data identify PVT as a central stress sensor in non-Pavlovian threat-related behavioral plasticity, in addition to its role in Pavlovian fear [26, 28, 46].

We next examined the downstream targets that could mediate stress-induced behavioral effects. In this regard, BLA-CE circuitry, being recruited during behavioral challenge itself (LA + BA and CE in Fig. 1d) and an immediate downstream target of PVT, emerged as a particularly good candidate. Viral injection of GFP-tagged AAV confirmed the CEl as primary PVT target (Supplementary Fig. S4a) [24]. In turn, the PVT provided the major source of afferent projections to the CEl (Supplementary Fig. S5). Combining CEl injections of the retrograde tracer Cholera toxin B (CTB) with c-fos activity mapping (Fig. 2c, left), revealed an increased fraction of c-fos^+^ in CTB^+^ PVT cells after exposure to EPM B, which was further potentiated by shock exposure (Fig. 2c, right). These results demonstrate that the PVT to CEl projection is also recruited by shock and behavioral challenge (cf. Fig. 1d) [35, 47].

Projection-specific bilateral optogenetic manipulation by stereoselective injection of AAV expressing ChR2, Arch or GFP and fiber stimulation of CEl (Supplementary Fig. S4a) further showed that activation of this projection during EPM A mimicked shock experience, whereas inactivation of the projection during EPM B tends to counteract the shock-induced switch to passive behaviors (Fig. 2d). Inactivation in EPM A and activation in EPM B did not show significant effects (data not shown). Thus, in the context of our paradigm, PVT-CEl projections seem primarily involved in relaying stress signals to CEl.

**Fig. 3:**
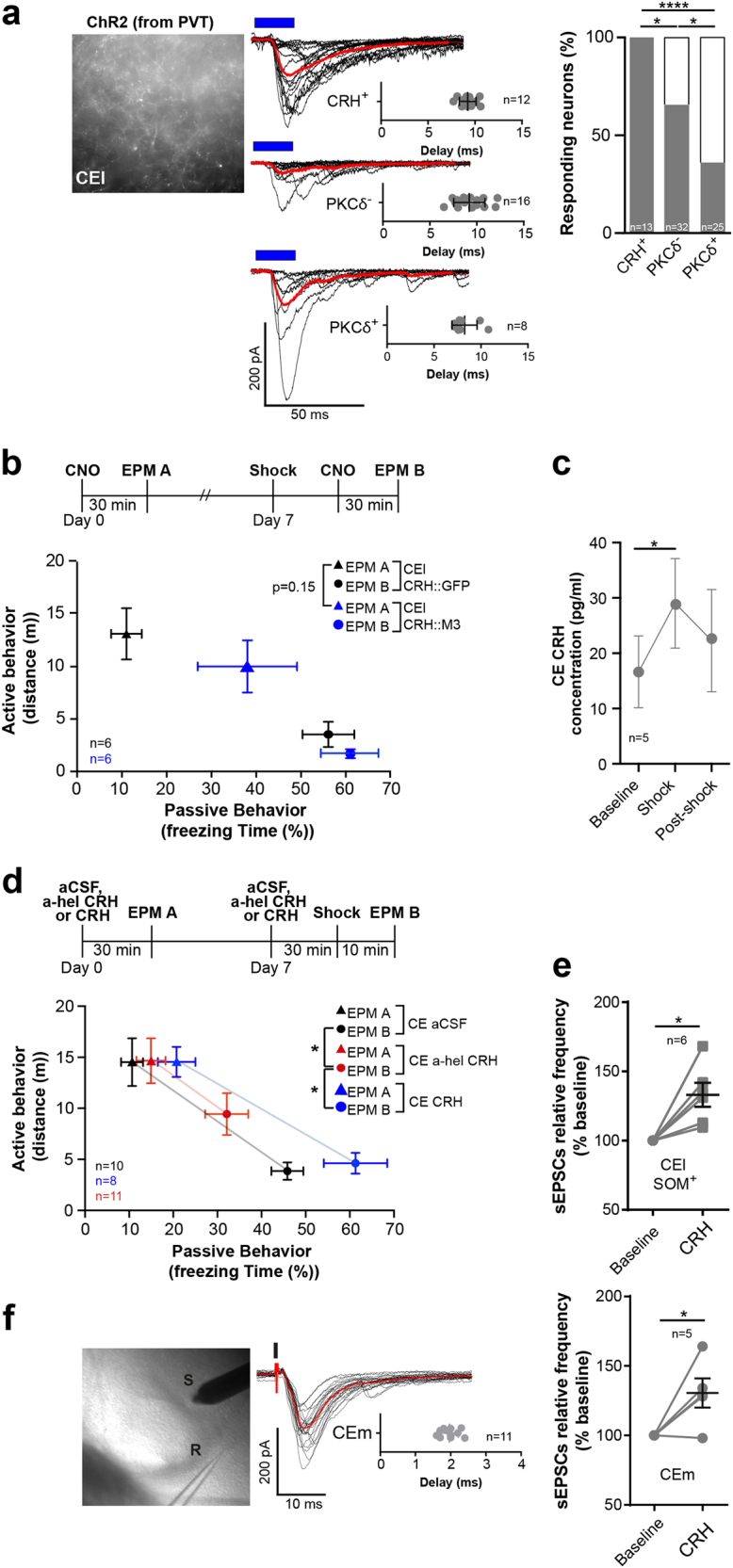
Stress and PVT interact with CE CRH to modulate passive responding. **a** Left, PVT terminals in CEl after ChR2 injection in PVT. Middle, genetically guided whole cell patch recordings of postsynaptic responses in CEl after 10 ms optogenetic stimulation (blue bars) of PVT terminals in CEl. Red, population averages of responding cells. Insets show response delays from onset of optogenetic stimulation. Right, population-specific innervation of CRH^+^ > PKCδ^-^ > PKCδ^+^ neurons in CEl (Fisher’s exact test *p* < 0.0001). PKCδ^+^, SOM^+^, and CRH^+^ cells represent the major neuronal types in CEl. PKCδ^−^ neurons correspond largely to SOM^+^ and CRH^+^ cells. **b** Top, experimental design. CNO was injected 30 min prior challenge exposure. Bottom, pharmacogenetic manipulation of CEl CRH^+^ cells during behavioral challenge. Activation of CRH^+^ cells increases passive behaviors without shock exposure (MANOVA *p* < 0.0001, Hotelling’s T^2^ post hoc tests). **c** Shock-induced CRH in CEl microdialysates from lightly anaesthetized animals (RM one-way ANOVA *p* = 0.03, Holm−Sidak post hoc test). **d** CE CRH signaling mediates behavioral modulation by stress experience. Top, timeline for intracerebral drug administration of aCSF, a-hel CRH or CRH in CEl. Bottom, a-hel CRH decreases passive behaviors during EPM B (MANOVA *p* < 0.0001, Hotelling’s T^2^). **e** Relative frequency of EPSCs in whole cell patch recordings of genetically tagged CEl SOM^+^ neurons after bath application of CRH (paired *t* test *p* = 0.0035). **f** CRH modulation of CEm neurons. Left, electrical stimulation of BLA (S) and whole cell patch recording (R) from neurons in CEm. Middle, responses of neurons after electrical stimulation (black bar). Red, cell average of a responding cell. Inset depicts response delay from onset of electrical stimulation. Right, relative frequency of EPSCs in whole cell patch recordings of CEm that are monosynaptically innvervated from BLA (paired *t* test *p* = 0.0433). Significance levels are given as *p < 0.05, **p < 0.01, ***p < 0.001, ****p < 0.0001

The CEl consists of several classes of antagonistic inhibitory neuronal populations marked by the expression of different proteins and peptides [7, 10, 11]. Optogenetic circuit mapping in brain slices of PKCδ::Cre transgenic animals injected with AAV for Cre-dependent GFP tagging and targeted electrophysiology revealed that PVT preferentially innervated PKCδ^-^ cells, a population largely consisting of CRH^+^ and SOM^+^ neurons [9, 13] (Fig. 3a). In an analogous experiment in CRH::Cre animals, virtually all CRH^+^ cells responded to PVT activation, indicating that PVT preferentially and monosynaptically projects to CRH^+^ > SOM^+^ > PKCδ^+^ neuronal populations (Fig. 3a).

The strong connectivity between PVT and CEl CRH^+^ neurons may link PVT stress signals to behavioral choice in CE. In this case, activating CEl CRH^+^ neurons should phenocopy the effects of PVT-to-CEl activation (Fig. 2d). Consistent with this notion, injecting CNO prior to EPM A exposure in animals virally expressing hM3Dq DREADD (designer receptors exclusively activated by designer drugs) for pharmacogenetic activation of CEl CRH^+^ cells increased passive behaviors in EPM A without shock (Fig. 3b). Interestingly, post-shock injection of CNO in animals virally expressing hM4Di DREADD for pharmacogenetic inactivation of CEl CRH^+^ cells alone was not sufficient to prevent switching to passive responding in EPM B (data not shown). Taken together, the primary role of PVT-to-CEl CRH^+^ interaction seems to convert shock signals to CEl CRH.

Since shock strongly activates PVT, which then primarily targets CEl CRH^+^ cells, we hypothesized that CRH should be locally released in CE in response to shock experience. We tested this by microdialysis (Supplementary Fig. S6) in lightly anaesthetized mice which revealed local shock-induced release of CRH (Fig. 3c), concomitant to increased tonic firing in PVT (Supplementary Fig. S7). Moreover, intra-CE infusion of local a-helical CRH, a corticotropin-releasing hormone receptor 1 (CRHR1) antagonist, impaired the switch from active to passive behaviors during EPM B (Fig. 3d), while having no effect during EPM A. Conversely, local infusion of CRH potentiated the shock-induced switch to passive behaviors during EPM B (Fig. 3d). The fact that CRH manipulation during EPM A alone was not effective enough to mimic shock experience suggests that increased activity of PVT (Supplementary Fig. S2b, Fig. 2c) or of other glutamatergic inputs and/or additional local mechanisms in CE contribute to shock-induced switching to passive responding.

At the cellular level, CRH increased the frequency of excitatory postsynaptic spontaneous currents (EPSCs) in CE neurons in slice electrophysiological recordings, consistent with a presynaptic potentiation of glutamatergic inputs to CE. Importantly, CRH increased glutamatergic inputs to two cell types with a strong link to passive responding: CEl SOM^+^ cells, which have the capacity to control passive (freezing) behaviors [7] (Fig. 3e, albeit not limited to this cell type in CEl, Supplementary Fig. S8c) and CEm neurons innervated by BLA (Fig. 3f). Both of these cell types receive neuromodulatory inputs from CEl CRH neurons ([9, 13] and Supplementary Fig. S8d).

**Fig. 4:**
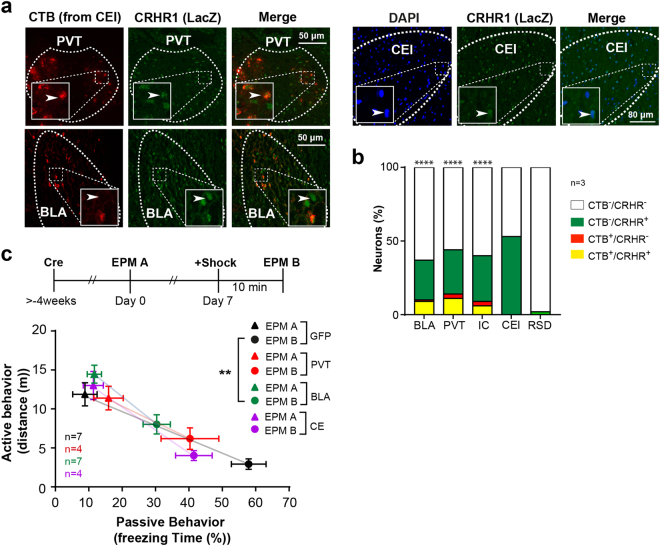
CRHR1 receptors facilitate stress-induced passive responding. **a** Expression of CRHR1 in CE afferents backlabelled with Cholera toxin B (CTB) and in CEl in CRHR1-tau-lacZ mice. CRHR1 expression was visualized with anti-lacZ IHC. **b** Quantification of CRHR1 expression (**a**) from three different animals. Inputs associated with stress-behavior (PVT and BLA) express high level of CRHR1 (Fisher’s exact tests for overlap of CTB and CRHR1). BLA basolateral amygdala, PVT paraventricular nucleus of the thalamus, IC insular cortex, RSD retrosplenial dysgranular cortex. **c** Region selective knock out of CRHR1 in PVT/BLA-CE circuitry. Experimental groups were injected with AAV::Cre into either PVT, BLA or CE and compared to controls injected with AAV::GFP into these regions (MANOVA *p* = 0.02, Hotelling’s T^2^ post hoc tests). Significance levels are given as *p < 0.05, **p < 0.01, ***p < 0.001, ****p < 0.0001

While the glutamatergic projection of PVT to CE was an obvious candidate, the fact that post shock perturbation of PVT-CE only partially reverted shock experience (Fig. 2d) suggested that additional glutamatergic inputs might be required for CRH-dependent behavioral plasticity. Among the major glutamatergic inputs to CEl (Fig. 4a), BLA showed the highest fraction of CRHR1^+^ projections (Fig. 4b, yellow vs. red fraction) suggesting that these inputs might be particularly susceptible to potentiation by CRH. This notion is supported by the facts that optogenetic manipulation of BLA-to-CEm projections modulates anxiety [37] and active/passive responding in EPM challenge (Supplementary Fig. S9). To investigate this functionally, we performed a region-specific CRHR1 knock-out in PVT, BLA and CE by injecting AAV::Cre expressing virus into homozygous floxed CRHR1^loxP/loxP^ animals (Supplementary Fig. S10).

Regarding behavioral switch in EPM B, local CRHR1 knock-out in the BLA showed the strongest effect, significantly reverting the stress-induced behavioral passive response bias (Fig. 4c), while EPM A behavior was unaffected. Knocking out CRHR1 in PVT or CE leads to a trend in the same direction (Fig. 4c). This is in line with the notion that the anxiety-related behavioral effects were dominated by presynaptic effects.

Taken together, our results put forward a model in which stress-driven activation of PVT-CEl CRH^+^ pathway potentiates BLA-to-CEm signaling via a CRH-dependent presynaptic mechanism. This direct activation might synergize with CRH effects on CEl SOM^+^ cells, which may antagonize inhibitory gating through local inhibition of PKCδ^+^ cells.

**Fig. 5:**
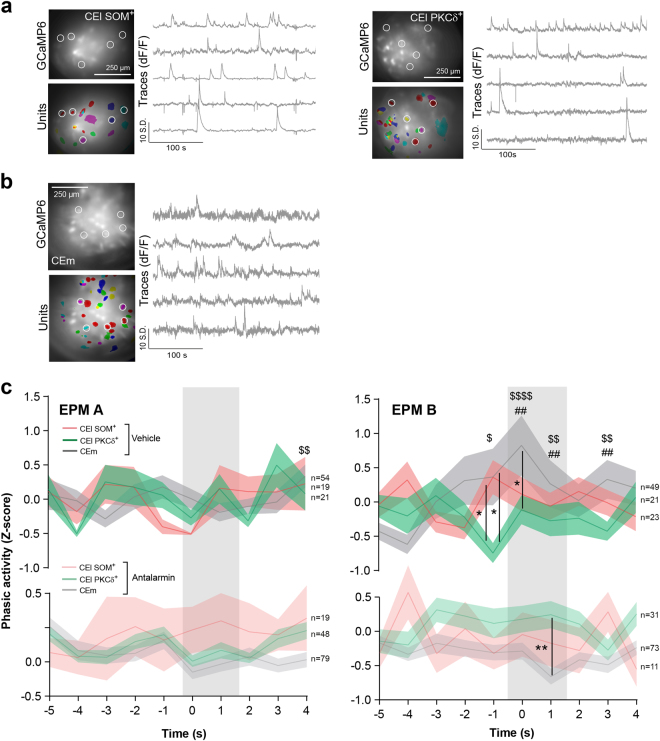
Stress and CRH modulate CE microcircuit interactions. **a**, **b** Deep brain calcium imaging of CEl and CEm in freely moving mice. Deep brain images of CEl SOM^+^, PKCδ^+^ and putative CEm neurons expressing GCaMP6 (top, average projection), individual isolated units (bottom) and example trace (right) of an isolate units (white circles in left panels). Note that in the average projections not all active units are visible. **c** Phasic activity of CEl and putative CEm neurons during behavioral challenge in EPM A or EPM B. Mean population activity during freezing episodes. After stress experience (EPM B) putative CEm activity was coupled to freezing onset, an effect reversed by application of antalarmin (RM two-way ANOVA *p*_interaction_ < 0.0001, Benjamini−Krieger−Yekutieli two-stage linear step-up post hoc test) (*n* indicates neurons extracted from 1 to 4 animals). These freezing-related responses  of CEl SOM^+^, PKCδ^+^ and putative CEm neurons resemble On-/Off-responses to Pavlovian fear cues [10, 11]. The opposite patterns of CEl PKCδ^+^ and CEm cell phasic activity may reflect the modulation of CEl-to-CEm inhibitiory gating by shock and antalarmin. The gray bars represent the first 2 s of freezing. *Between cell type comparisons. # Within cell type comparisons to EPM A. $ Within cell type comparison to antalarmin treatment. Significance levels are given as */#/$ *p* < 0.05, **/##/$$ *p* < 0.01, ***/###/$$$ *p* < 0.001, ****/####/$$$$ *p* < 0.0001

To support this hypothesis, we explored CRH-dependent signatures of shock experience in CE circuit dynamics. To this end, we performed deep brain Ca^2+^ imaging of SOM^+^ and PKCδ^+^ neurons in CEl (Materials and Methods, Supplementary Fig. S11a, Fig. 5a) and putative CEm neurons (Materials and Methods, Supplementary Fig. S11b, Fig. 5b) which represent partially antagonistic and functionally distinct components of CE fear circuitry [10, 11, 37]. We reasoned that shock experience and CRH should antagonize inhibitory gating of CEm output by CEl PKCδ^+^ cells and, in consequence, increase the phasic coupling between CEm activity and passive behavioral responding.

Indeed, shock experience increased the correlation between phasic activity in putative CEm neurons and freezing (Fig. 5c, Supplementary Fig. S12b), whereas CEl PKCδ^+^ cells, but not CEl SOM^+^ neurons, showed the opposite effect (Fig. 5c, Supplementary Fig. S12b). The antagonistic phasic behavior of CEl and CEm after shock resembles circuit dynamics underlying Pavlovian fear conditioning, where neurons in CEl (potentially PKCδ^+^ cells) and CEm have opposite roles [10, 11]. Note that the tonic activity was not significantly different across these conditions (Supplementary Fig. S12a).

These shock-induced modulations of phasic response patterns were suppressed by systemic administration of antalarmin (Fig. 5c), a CRHR1 antagonist that reduces anxiety in EPM [48], blocks stress-induced anxiety [49] and abolished the switch to passive behavior in our assay (Supplementary Fig. S13). Antalarmin reverted the stress-induced phasic coupling of putative CEm neurons’ activity with freezing (Fig. 5c).

Collectively, the imaging data suggest that stress and CRH antagonize inhibitory gating by CEl (potentially PKCδ^+^ neurons therein) while facilitating CEm activity and freezing.

## Discussion

Despite its clinical relevance, our understanding of the neural mechanisms underlying stress-induced behavioral adaptations is rather limited. To address this problem, we studied the effects of inescapable shock on exploratory behavior in an EPM maze [19, 20, 45]. In this assay stress experience, while increasing anxiety (Supplementary Fig. S1a, c) leads to a pronounced shift from active exploration to passive freezing (Fig. 1c). Thus, it represents a simple method for monitoring stress effects on active/passive behaviors when coping with behavioral challenge, in a non-Pavlovian, anxiety-related setting. While complex paradigms (e.g. active avoidance) address coping more directly, we believe that our findings can be interpreted within the context of stress-related passive avoidance and emotional coping in humans.

Based on functional experiments in this assay, we propose a model in which the stress peptide CRH sensitizes a canonical fear circuit to promote passive behavioral responding (Supplementary Fig. S14). In this model, stress activates PVT projections to CEl CRH^+^ neurons. Locally released CRH facilitates glutamatergic signaling at BLA inputs to cell types in CE associated with passive responding (CEl SOM^+^ neurons [23] and CEm output). This, in turn, antagonizes inhibitory gating of CEm by CEl PKCδ^+^ neurons while facilitating BLA-CEm connectivity to promote freezing.

The routing of behavioral challenge through BLA and stress experience through PVT reflects two lines of results. BLA was recruited by EPM challenge but not potentiated by shock (LA/BA in Fig. 1d). PVT was potentiated by shock (Fig. 1e) and PVT-CE connections relayed shock (Fig. 2c, d) but were only partially required for shock effects in EPM B (Fig. 2d).

The fact that PVT-CE CRH heterosynaptically potentiates BLA−CE connectivity explains that PVT-CE (Fig. 2d) as well as CE CRH^+^ neurons (trend in Fig. 3b) when activated, mimicked shock in EPM A, but contributed only partially to post-shock behavior in EPM B (Fig. 2d and data not shown). The heterosynaptic interaction is also supported by the observation that CRH (Fig. 3d), BLA-CEm connection (trend in Supplementary Fig. S9) and BLA CRHR1 (Fig. 4c) were necessary for stress modulation in EPM B. However, we have currently no definite explanation for why CE CRH infusions were not sufficient to mimic shock in EPM A (Fig. 3d). The fact that they seemed to exacerbate shock experience in EPM B points to the synergistic effects of increased tonic activity in PVT inputs (Supplementary Fig. S2b) which preferentially targets CRH^+^/SOM^+^ but not PKCδ^+^ cells (Fig. 3a)(Supplementary Fig. S14).

Comparing neuronal activity in PVT, CEl and putative CEm neurons during behavioral challenge revealed that PVT, while phasically active during shock (Fig. 1e), was only tonically modulated in EPM (Supplementary Fig. S2b) and uncoupled from freezing (Supplementary Fig. S2d). On the other hand, after stress, CE couples strongly with freezing (Fig. 5c). These different activity patterns reflect the underlying functions of PVT cells in relaying tonic stress signals and BLA-CEm controlling phasic active/passive behavioral choices.

Our study complements recent findings in which PVT/BLA−CE circuit interactions [10, 11, 28, 36] and CE CRH signaling in SOM^+^ neurons [9] have been implicated in modulating Pavlovian fear. Recent seemingly contradictory findings on the specific role and mechanism of CEl PKCδ^+^ [11, 15] or CEl SOM^+^ [9, 13] cells in modulating Pavlovian fear indicate that future studies on CE neuronal function and dynamics are clearly needed. Interestingly, even with the data at hand, the phasic responses of CEl PKCδ^+^ and putative CEm neurons phenotypically resemble CE microcircuit dynamics in Pavlovian fear. In this case, CEl [50] (and potentially CEl SOM^+^ vs. PKCδ^+^ cells therein [10, 11]) and CEm are also oppositely responding to fear cues. Moreover, different neuronal types in the CE, in particular Nrip2^+^ [12], SOM^+^ [23] and CRH^+^ [13], control active/passive fear responses. Thus, PVT/BLA-CE circuitry seems to multiplex active/passive behavioral decisions in Pavlovian and non-Pavlovian behavioral plasticity by shared elements (PVT-CE-CRH^+^ and BLA-CEl SOM^+^/CEm circuits) and dynamic states (inhibitory gating of CEm output by CEl) to control active/passive responding.

However, this circuitry relies on different cellular mechanisms to mediate Pavlovian fear and non-Pavlovian plasticity. Our data show that in a non-Pavlovian anxiety-related setting, CRH-dependent modulation of BLA−CE connectivity was mediated by presynaptic effects (Figs. 3e, f, 4c), which might, in part, synergize with CRH-driven postsynaptic facilitation of BLA inputs to CEl SOM^+^ cells [9].

In contrast, Pavlovian fear evokes long-term synaptic plasticity, that relies in part on postsynaptic effects at BLA-CE synapses [7], which are gated also by CRH [9]. Interestingly, while presynaptic CRH effects at BLA-CE synapses are largely autonomous, CRH effects on postsynaptic long-term potentiation are facilitated by dopamine (DA) [40]. As amygdala DA signaling gates Pavlovian processes [45, 51], non-Pavlovian and Pavlovian plasticity are dissociated by pre- and postsynaptic mechanism at the same sites by DA. Given that CRH primes both mechanisms, this might provide a mechanism for reinforcement of anxiety, fear learning and a bias for passive responding.

The model (Supplementary Fig. S14) poses that CRH sensitizes BLA−CE interactions and lowers the response threshold for passive behaviors to aversive stimuli in behavioral challenge. The EPM, like any anxiogenic maze, naturally suppresses exploration. In our case, this increased sensitivity, visible as reduced exploration and increased passive freezing, is exacerbated by shock-experience and CRH. Likewise, CRH also increases passive responding to weak threats in Pavlovian settings [9]. Interestingly, the CEl (gated internally by CEl Nrip^2+^ cells) also modulates active/passive fear through interacting with basal forebrain cholinergic signaling by modulating arousal [12]. Thus, CRH sensitizes passive responding by facilitating BLA-CEm signaling and antagonizes arousal and active behaviors by CEl−forebrain interactions. Dissociating these ethological dimensions (active/passive vs. arousal) in PVT-amygdala circuitry will be an interesting avenue for future research.

Paradoxically, CEl CRH^+^ cells also facilitate active responding in cases where active fear behavior is the appropriate conditioned response [13]. One possible explanation is that in low intensity settings CEl CRH^+^ cells increase freezing by CRH and facilitate BLA inputs to CEl SOM^+^/CEm neurons. In high anxiety settings, this effect is overridden by direct local GABAergic inhibition between CEl CRH^+^ and CEl SOM^+^ cells. Thus, CEl CRH^+^ neurons might control the switch from active exploration to passive freezing ([9, 12] and this study) and freezing to active escape [13] by different neurotransmitters.

From a translational perspective, the convergence of Pavlovian and non-Pavlovian plasticity in PVT/BLA-CE dynamics might explain some of the mutual reinforcement of stress, anxiety and Pavlovian fear [52, 53]. Moreover, it provides a potential mechanism for the passive coping bias observed in GAD and PTSD in humans [18, 21, 54, 55]. Lastly, our study identified sites through which CRHR1 controls anxiety [56] and provides a neuronal mechanism for the anxiolytic properties of CRHR1-directed therapeutics [57].

## Electronic supplementary material

Supplemental Material
