# Supplementary material for: Stress peptides sensitize fear circuitry to promote passive coping
Source: Mol Psychiatry. 2018 Jun 14;25(2):428–41. doi: 10.1038/s41380-018-0089-2 (PMC6169733; doi:10.1038/s41380-018-0089-2)
Supplement: Supplementary file 1 — Supplemental Material [file 41380_2018_89_MOESM1_ESM.docx]

**SUPPLEMENTARY EXPERIMENTAL PROCEDURES**

**Surgery**

The surgical protocol was adapted from Athos and Storm ^1^. All mice were between 8-12 weeks when surgery started. Mice were deeply anaesthetized with isoflurane (5%, Isoflo, Abbot Laboratories) until the absence of plantar reflexes and placed on a stereotaxic alignment apparatus (Model 1900, David Kopf Instruments). The head was mounted in the stereotaxic frame, positioned within the anesthesia nosepiece and supplied with isoflurane 1.6-2%. The skull was then attached to nonrupturing ear bars (Model 1922, David Kopf Instruments). A heating pad controlled by a rectal thermometer (DC temperature controller (FCH) was used to maintain body temperature at 36 ºC. 0.1 ml of Lidocaine (Xylanaest 1%, Gebro Pharma) was injected subcutaneously prior to exposing the skull. An incision was made in the anterior/posterior plane to expose the skull. The stereotaxic alignment indicator (Model 1905, David Kopf Instruments) was used to adjust the arm position along the lateral and anterior-posterior axis. After the alignment, bregma was located and used as the standardized landmark. All stereotaxic coordinates were calculated relative to bregma according to the mouse Atlas ^2^. The skull was drilled with a stereotaxic mounted drill (Model 1911, David Kopf Instruments) at the desired coordinates.

At the end of the surgery, the skin was sutured, and the mouse was positioned on a heat-map inside a clean cage until full recovery. Approximately two hours later, the mouse returned to the home cage, where it stayed grouped-housed. For postoperative care, drinking water was supplied with Carprofen (Rimadyl, Pfizer, 250 mg/l) and Enrofloxacine (Baitryl, KVP pharma, 400 mg/ml) for 10-14 days.

Injection-coordinates relative to bregma (mm). All CEl injections and optogenetic cannula placements were bilateral. Ca^2+^ imaging microendoscopes were implanted unilaterally in the right hemisphere around 0.2 mm above the injection site.

| **Brain Region** | **Bregma** | **Lateral** | **Ventral** |
| --- | --- | --- | --- |
| CEl | -1.35 | 2.85 | 4.8 |
| CEm | -1.06 | 2.25 | 4.6 |
| (posterior) PVT | -1.58 | 0 | 3.4 |
| BLA | -1.58 | 3.3 | -5.1 |

The Mirco4 Syringe Pump Controller (World Precision Instruments) was used for all injections to regulate collection and injection volumes. Injection rate was set to 50 nl/min. After the injection was completed, the glass needle was left in place for supplemental 5-10 min to guarantee complete injection and diffusion of the solution. For CTB injections, CTB-Alexa Fluor-555 (C-22843, Invitrogen) was used, NMDA was acquired from Sigma-Aldrich, 50 mg/ml in PBS.

| **Virus** | **Source** | **Titer(GC/ml)** |
| --- | --- | --- |
| AAV9.hsyn.GCaMP6m.WPRE | Internal | 1.0 x 10^13^ |
| AAV1.Syn.Flex.GCaMP6f.WPRE.SV40 AV-1-PV2819 | University of Pennsylvania | 1 x 1013 |
| AAV2/5.hsyn.hChR2(H134R).eYFP.WPRE (Addgene 26973P) | University of Pennsylvania | 1.2 x 10^13^ |
| AAV5.hsyn.ArchT.YFP.WPRE.hGH | University of Pennsylvania | 4.7 x 10^12^ |
| AAV2/5.hSyn.DIO.hCHRM3.mCherry.WPRE.hGh | University of Pennsylvania | 2.1 x 10^12^ |
| AAV2/5.EF1a.DIO.GFP.WPRE | Internal | 2.1 x 10^12^ |
| AAV5.hsyn.eGFP.WPRE.hGH | University of Pennsylvania | 1. 15E+13 9.8 x 10^10^ |
| AAV5.Cre | Vectorbiolabs | 1 x 10^10^ |

For cannulations, the optogenetic fiber optic cannulas (Doric Lenses, MFC 200/245-0.375 mm ZF1.25 FLT) or guide cannulas for injections (Bilaney Consultants GmbH, C313GS) were implanted 0.5 mm above the ventral position indicated previously and stabilized with dental cement (SuperBond C&B kit with L-Polymer, Prestige Dental Products Ltd).

**Ex vivo electrophysiology**

For the preparation of brain slices, 2-3 months old transgenic SOM::Cre, PKCδ::Cre and CRH::Cre male mice, previously injected either with AAV5 for expression of RFP-tagged ChR2 (for projection specific activation) and/or Cre-dependent GFP expression (AAV2/5.EF1a.DIO.GFP.WPRE for genetically guided electrophysiology) with <100 nl at 1x10^12^ U/ml in PBS, were used. These mice were deeply anesthetized with Isoflurane, decapitated and their brains quickly chilled in sucrose-based dissection buffer (220 mM sucrose, 26 mM NaHCO_3_ , 2.4 mM KCl, 10 mM MgSO_4_, 0.5 mM CaCl_2_ , 3 mM sodium pyruvate, 5 mM sodium ascorbate and 10 mM glucose), bubbled with 95% O_2_/5% CO_2_. Brain slices (300 μm) were cut in dissection buffer using a vibratome (Leica, VT1000S) and immediately incubated for 15min recovery phase in oxygenated aCSF in 95% O_2_/5% CO_2_ at 32 °C. This was followed by a slice-resting phase with oxygenated aCSF (126 mM NaCl, 2.5 mM KCl, 1.25 mM NaH_2_PO_4_, 26 mM NaHCO_3_, 2.5 mM CaCl_2_, 2.5 mM MgCl_2_, and 25 mM glucose) for at least 45 min at room temperature.

For electrophysiological recordings, individual brain slices containing the area of CEl were placed on the stage of an upright, infrared-differential interference contrast microscope (Olympus BX50WI) mounted on an X-Y table and visualized with a 40x water immersion objective by an infrared sensitive digital camera (Hamamatsu, ORCA-03). Slices were entirely submerged and continuously perfused at a rate of 1-2 ml per min with oxygenated aCSF. CRH^+^, PKCδ^+^ and SOM^+^ neurons in the CEl were identified by the presence of injected GFP expressing virus. Patch pipettes were pulled on a Flaming/Brown micropipette puller (Sutter, P-97) from borosilicate glass (1.5 mm outer and 0.86 mm inner diameter, Sutter) to final resistances ranging from 3 to 5 MΩ. Membrane currents were recorded with a Multiclamp 700B amplifier (Molecular Devices). Electrophysiological signals were low-pass filtered at 3 kHz, sampled at 10 kHz and stored on a PC for offline analysis with pClamp 10 software.

For the optogenetic manipulations during electrophysiological recordings, inputs from PVT to CEl were stimulated in voltage-clamp (-70 mV) with 20 ms blue light pulses through a 40x electrophysiology microscope objective, driven by a 120 W mercury lamp (X-Cite 120 PC Q). The amplitude of 4 pulses, 1 second apart, was averaged as postsynaptic responses of specific cell types in the CEl. Internal solution contained (in mM): 135 mM cesium methanesulfonate, 5 mM KCl, 10 mM HEPES, 2 mM MgCl_2_, 0.2 mM EGTA, 1 mM Na_2_ATP, 0.4 mM NaGTP, 10 mM Na_2_Phosphocreatine, 280-290 mOsmol

For the CRH application experiments, 5 minutes after allowing the cell to reestablish constant activity post break-in, whole-cell voltage-clamp recordings of AMPA receptor-mediated sEPSCs, isolated by adding Bicuculline (10 µM, Sigma), were baseline recorded for 4 minutes in voltage clamp (-70 mV), followed by application of CRH 1 μM (Sigma) to the bath. Internal solution contained: 135 mM K-gluconate, 5 mM KCl, 10 mM HEPES, 2 mM MgCl_2_, 0.2 mM EGTA, 1 mM Na_2_ATP, 0.4 mM NaGTP, 10 mM Na_2_Phosphocreatine.

**In vivo electrophysiology recordings in anaesthetized mice and analysis**

Surgeries were conducted as previously described ^3,4^, with some modifications. On the day of the experiment, mice were anaesthetized with isoflurane followed by injection of a ketamine/xylazine mix (17 mg/kg and 7 mg/kg, respectively; Ketasol, OGRIS Pharma Vertriebs-GmbH, Xylasol, OGRIS Pharma Vertriebs-GmbH) and mounted on a stereotactic alignment apparatus (Model 963, David Kopf Instruments). For the duration of the experiment, supplemental intraperitoneal administration of the mix was provided as needed to maintain anesthesia and suppress the hindlimb compression reflex. The body temperature was monitored and maintained at 36^o^ C with a heating pad. The fur was shaved and, following an incision over the scalp, the skull was exposed. Two holes were drilled in the left and right occipital bones and two screws implanted which served as reference and ground, respectively. A third burr hole was drilled overlying the site of recording, the PVT (coordinates described previously), to expose the cortical surface. A 16-channel linear silicon probe (Neuronexus) was slowly lowered into the PVT; the cortical surface was kept moist with saline for the entire duration of the experiment. Recording sites were connected through a 16-channel headstage (Plexon) to a computer-controlled preamplifier (Digital Headstage Processor, Plexon, gain 100×, bandpass filter from 150 Hz to 9 kHz) and continuously digitized at 40 kHz. The resulted signal was fed into a multichannel amplifier and neural data acquisition system (OmniPlex, Plexon), isolated in WB frequencies (bandpass filter from 250 Hz to 8 kHz) and units were discriminated on-line by template matching and window discrimination analysis (OmniPlex, Plexon).

After the probe stabilized for ~60 min, recording of neuronal activity began, and baseline activity was recorded for 20 min. Then recording continued during electrical stimulations of the sciatic nerve (shock; ^5^) with a protocol similar to the one employed in the behavioral experiments (0.5-1 mA, 1-sec; randomly repeated every ~1-min for 10 consecutive times). Repeated shocks were delivered to the footpad contralateral to the recording site via an animal shocker (Precision regulated animal shocker H13-15 (Coulbourn Instruments)) controlled by an Arduino and triggering a signal to the analog-input channel of the OmniPlex data acquisition system for off-line synchronized analysis. Post-shock neuronal activity was monitored and recorded for a minimum of 30 min. In two of the seven experiments, a second footshock was delivered and was followed by recording of post-shock activity. At the end of experiments, subjects were perfused, and the brains removed for standard histological verification of probe location.

Analysis of the recorded units was performed off-line with Off-Line Spike Sorter (Plexon). Footshock artifacts were removed manually, and units were extracted from unsorted waveforms by principal component analysis and a template-matching algorithm with Off-Line Spike Sorter, as previously described ^6^. Clusters with a clear separation were considered valid and acceptable if they met two criteria: 1. The J3 statistic - the ratio between between-cluster to within-cluster scatter and 2. The Davies–Bouldin validity index - the ratio of the sum of within-cluster scatter to between-cluster separation. Valid clusters were further inspected by the investigator in the 2D/3D space and by employing different tools provided by the software, which also identified spurious signals such as electrical noise ^6^. Waveforms from valid clusters were assigned to individual neurons on the basis of their vector cluster clouds, stability over time, autocorrelograms (clear refractory periods) and crosscorrelograms (no refractory periods).

The chronic effect of shocks was examined by analyzing the change in firing rate that occurred during shock delivery and the following period 10 min after shocks. A 95% CI of the baseline firing rate was calculated for each cell, and this parameter defined whether the change in firing rate after footshock was significant within each cell. In addition, a one-way ANOVA test assessed the significance of neuronal responses within groups.

**Microdialysis**

The guide cannulas were initially surgically implanted bilaterally (MBR-5, Bioanalytical Systems, Inc.). After at least two weeks of recovery, the mice were slightly anaesthetized with a Ketamine/Xylazine solution (Ketasol, OGRIS Pharma Vertriebs-GmbH, 10 mg/ml in PBS and Xylazine, Xylasol, OGRIS Pharma Vertriebs-GmbH, 1 mg/ml) and the microdialysis probe (MBR-1-5 Brain Microdialysis Probes (MD-2211, Bioanalytical Systems, Inc)) was inserted. The flow rate was 1μl/min and the samples were collected as follows: 10min prior shock, during the shock period (1mA, 1s, 10 shocks in 10min) and 10min post the shock, taking into account the delay between mouse and collection tube.

For detection of CRH, an ELISA-based method was used according to manufacturer’s instructions with detection range 12.35 - 1000 pg/ml (LS-F9544, LifeSpan BioSciences, Inc).

**Transcardial perfusion****, preparation of brain slices and immunochistochemistry**

Prior the procedure Ketamine/Xylazine (OGRIS Pharma Vertriebs-GmbH, 10 mg/ml in PBS, OGRIS Pharma Vertriebs-GmbH, 1 mg/ml) was administered intraperitoneally. The procedure was continued only when the plantar and ocular reflexes were completely absent. The mouse was then secured in a supine position by pinning the paws to a Styrofoam surface, and an incision was made to expose the thorax and the heart. Then, using a 21 G butterfly, the left ventricle of the heart was pinned, and the butterfly was inserted approximately 3mm and the right atrium was incised. Then, heparin solution (Sigma-Aldrich, 10 U/ml Heparin/PBS) was infused until the blood was washed out. 4 % PFA/PBS was flushed with a total volume of 30-40 ml depending on the perfusion performance (Paraformaldehyde (PFA) 4%). Mice were decapitated using scissors and three cuts were made on the cranium, the first along the mid-sagittal suture and two horizontally. The extracted brain was incubated with sucrose/PBS (Sucrose crystals, Fluka Biochemika, 15% Sucrose/PBS (m/V)) for dehydration and stored in 4 ºC overnight.

Brain slices were obtained by employing a cryostat protocol (Microm HM560). Extracted brains were embedded in a cryoprotective embedding medium (Tissue Tek, Sakura Finitech B.V.) contained in a plastic cryoblock, deeply frozen in dry ice and immersed in 96% ethanol for several minutes. When longer storage was needed, the embedded brains were placed at -80ºC until the cutting procedure. The fixed and frozen brains were sliced into 20 µm thick coronal sections on a cryostat. The slices were mounted onto microscopy slides (Thermo Scientific) and kept at -80ºC for further use.Cryosections (20 µm) were dried for at least 30 min at room temperature and then rehydrated and permeabilized in PBST for 10 min. For blocking of non-specific binding, the slides were incubated for 30 min in BSA. Primary antibodies (Anti-c-Fos (polyclonal IgG), ab7963, Abcam, Anti-PKCδ (IgG 2b), 610398, BD Biosciences, Anti-NeuN (monoclonal IgG 1), MAB377, Merck Millipore, GalZ, ab9361, Abcam) diluted 1:1000 in BSA (Sigma-Aldrich) were applied to the slides, and then incubated overnight at 4 ºC in a humidified chamber. The next day, the slides were rinsed three times in PBST for 10 min (TritonX-100, Sigma-Aldrich used as PBST (PBS plus 0.1% Triton X-100)). The secondary antibodies and DAPI (both 1:1000 dilution in BSA, Anti-Rabbit (IgG (H+L)), A-21202, Life technologies ®, Anti-Mouse (IgG (H+L)), A-21052, Life technologies ®, DAPI, D3571, Invitrogen) were applied to the slides. After incubation for 2 h in dark at room temperature the unbound secondary antibodies were washed out by washing in PBST three times for 10 min. Sections were mounted in Fluorescence Mounting Medium (Dako, S302380) and covered with coverslips to preserve for further analysis.

**SUPPLEMENTARY REFERENCES**

1 Athos, J. & Storm, D. R. High precision stereotaxic surgery in mice. *Current protocols in neuroscience / editorial board, Jacqueline N. Crawley ... [et al.]* **Appendix 4**, doi:10.1002/0471142301.nsa04as14 (2001).

2 Franklin, K. B. J. & Paxinos, G. *Paxinos and Franklin's The mouse brain in stereotaxic coordinates*. Fourth edition. edn, (2007).

3 Valenti, O. & Grace, A. A. Entorhinal cortex inhibits medial prefrontal cortex and modulates the activity states of electrophysiologically characterized pyramidal neurons in vivo. *Cerebral cortex* **19**, 658-674, doi:10.1093/cercor/bhn114 (2009).

4 Forro, T., Valenti, O., Lasztoczi, B. & Klausberger, T. Temporal organization of GABAergic interneurons in the intermediate CA1 hippocampus during network oscillations. *Cerebral cortex* **25**, 1228-1240, doi:10.1093/cercor/bht316 (2015).

5 Valenti, O., Lodge, D. J. & Grace, A. A. Aversive stimuli alter ventral tegmental area dopamine neuron activity via a common action in the ventral hippocampus. *The Journal of neuroscience : the official journal of the Society for Neuroscience* **31**, 4280-4289, doi:10.1523/JNEUROSCI.5310-10.2011 (2011).

6 Nicolelis, M. A., Dimitrov, D., Carmena, J. M., Crist, R., Lehew, G., Kralik, J. D. *et al.* Chronic, multisite, multielectrode recordings in macaque monkeys. *Proceedings of the National Academy of Sciences of the United States of America* **100**, 11041-11046, doi:10.1073/pnas.1934665100 (2003).

7 Sanford, C. A., Soden, M. E., Baird, M. A., Miller, S. M., Schulkin, J., Palmiter, R. D. *et al.* A Central Amygdala CRF Circuit Facilitates Learning about Weak Threats. *Neuron* **93**, 164-178, doi:10.1016/j.neuron.2016.11.034 (2017).

8 Haubensak, W., Kunwar, P. S., Cai, H., Ciocchi, S., Wall, N. R., Ponnusamy, R. *et al.* Genetic dissection of an amygdala microcircuit that gates conditioned fear. *Nature* **468**, 270-276, doi:nature09553. (2010).

9 Li, H., Penzo, M. A., Taniguchi, H., Kopec, C. D., Huang, Z. J. & Li, B. Experience-dependent modification of a central amygdala fear circuit. *Nature neuroscience* **16**, 332-339, doi:10.1038/nn.3322 (2013).


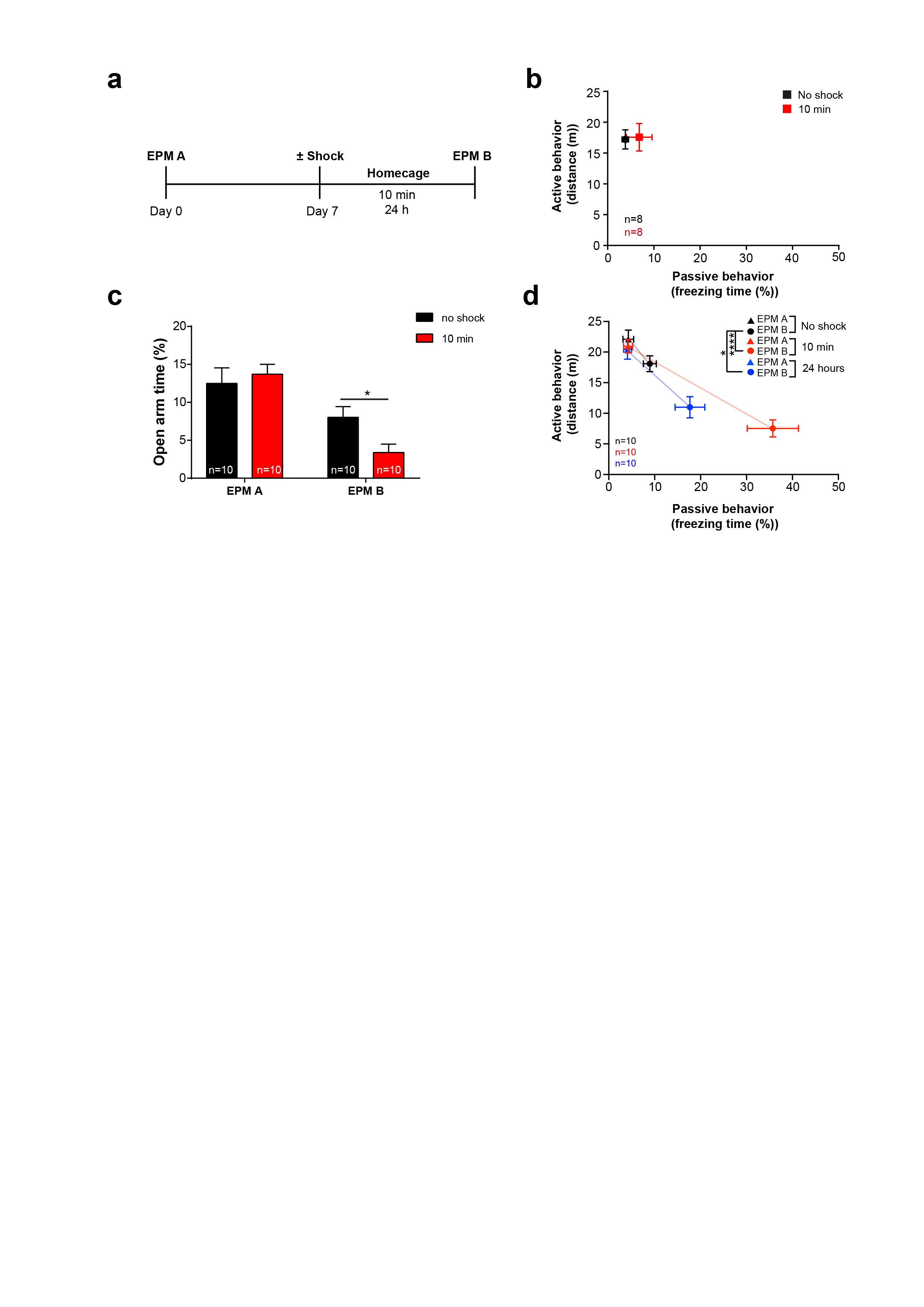


Supplementary Figure S1. Acute and long-term effects of stress on behavior. a Behavioral paradigm including time points that different groups were tested after shock-context exposure. b Quantification of active and passive behavior during exposure to homecage after the shock (MONOVA n.s.). c Quantification of anxiety measures in EPM A and EPM B. Shock reduces time spent in the open arms (RM two-way ANOVA p_Interaction_<0.01, Holm Sidak post hoc test) indicating slightly elevated anxiety in these animals. d Quantification of active and passive behavior reveals strong acute (10 min) and long-term (24 hours) persistent effects (MANOVA p<0.0001, Hotelling T^2^). Note that the behavioral changes during shock exposure (Figure 1b) revert in the home cage (b), but become apparent during behavioral EPM challenge (d). This indicates that behavioral changes observed during EPM B are not merely continued freezing states but rather reflect a shift in behavioral responding directed to EPM challenge. Comparing significance levels on open arm time (c) and active passive behaviors (d) indicates that shock experience slightly increase anxiety but strongly modulates behavioral choice. The different effect sizes 10 min or 24 h after shock might reflect acute and chronic components. This study focuses at the acute phase (10 min), when the effects are strongest. Significance levels are given as * p<0.05, ** p<0.01, *** p<0.001, **** p<0.0001.


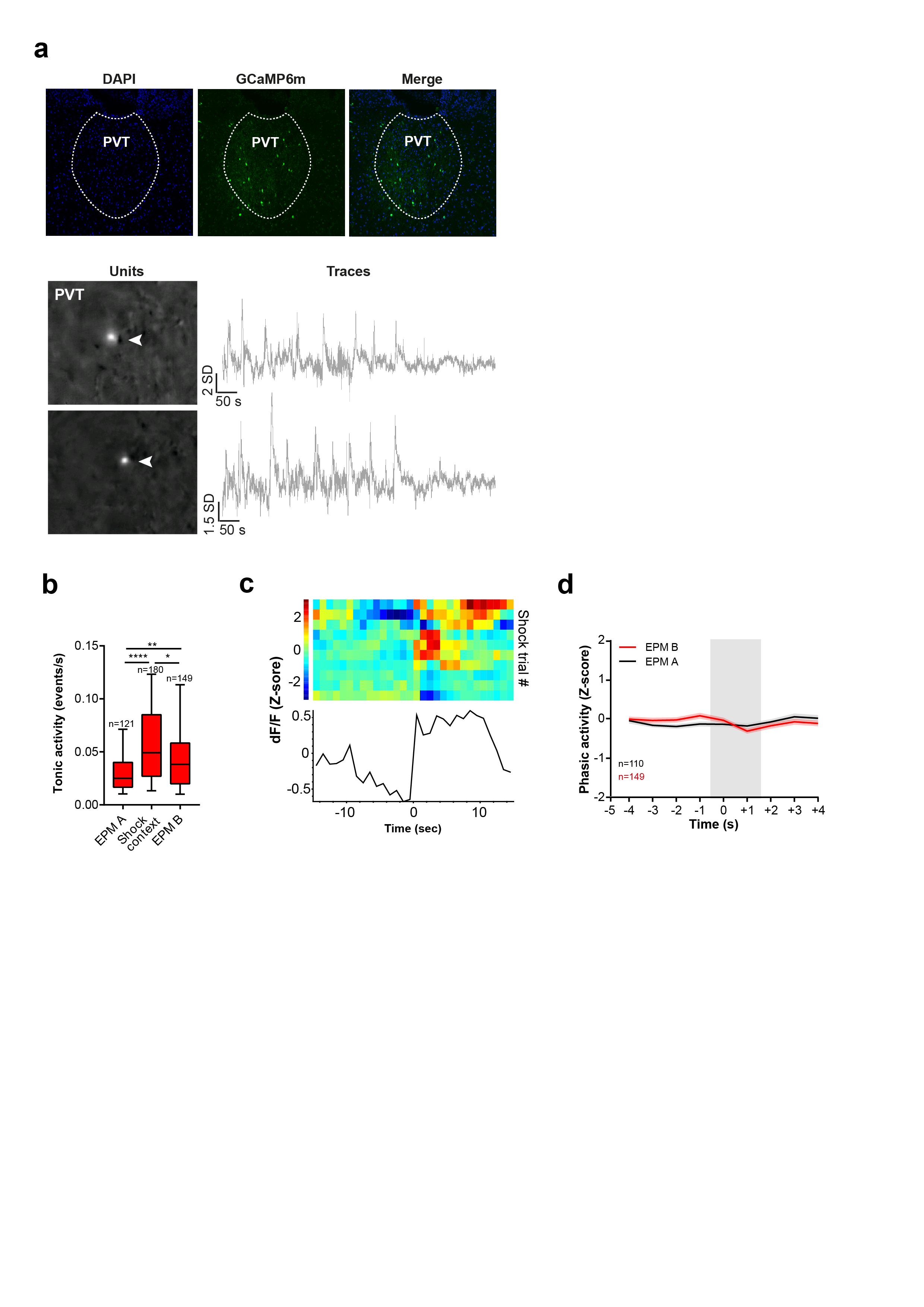


Supplementary Figure S2. PVT neuronal responses during shock context and behavioral challenge. a *Top*, GCaMP6m injections for calcium imaging were localized in posterior PVT. *Bottom,* Isolated units (arrowheads) and corresponding Ca^2+^ traces in behaving animals. b Mean population activity during EPM A, shock context and EPM B. Stress increases tonic activity of PVT during behavioral challenge (Kruskal-Wallis test p<0.0001, Dunn’s post hoc test) (n indicates neurons extracted from 5 animals). c Peri-shock histogram of Ca^2+^ signals of one unit with phasic shock responses. *Top*, peri-event activity during single freezing episodes, *bottom*, peri-event average. d PVT phasic activity coupling to freezing is not increased after shock experience. Phasic activity was calculated from Z-scores of events with the full recording session as base (RM two-way ANOVA_Time_ p=0.0669, Holm-Sidak post hoc tests). The gray bar represents the first 2 s of freezing. Significance levels are given as * p<0.05, ** p<0.01, *** p<0.001, **** p<0.0001.


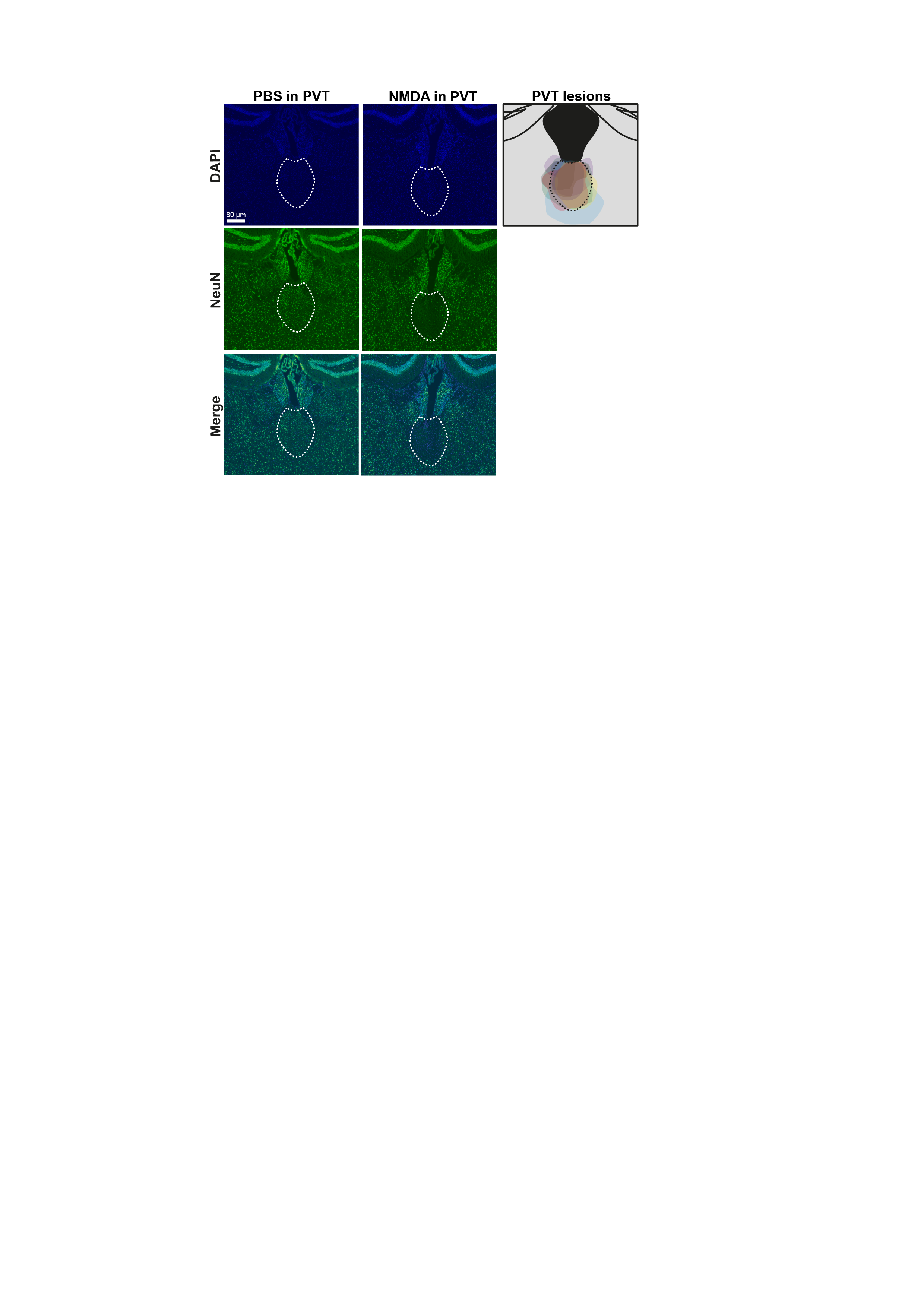


**Supplementary Figure S3.** Histological assessment of NMDA lesions. *Left,* Note the absence of the neuronal marker NeuN in the PVT after lesion. *Right*, Projections of the extent of lesions in the experimental group.


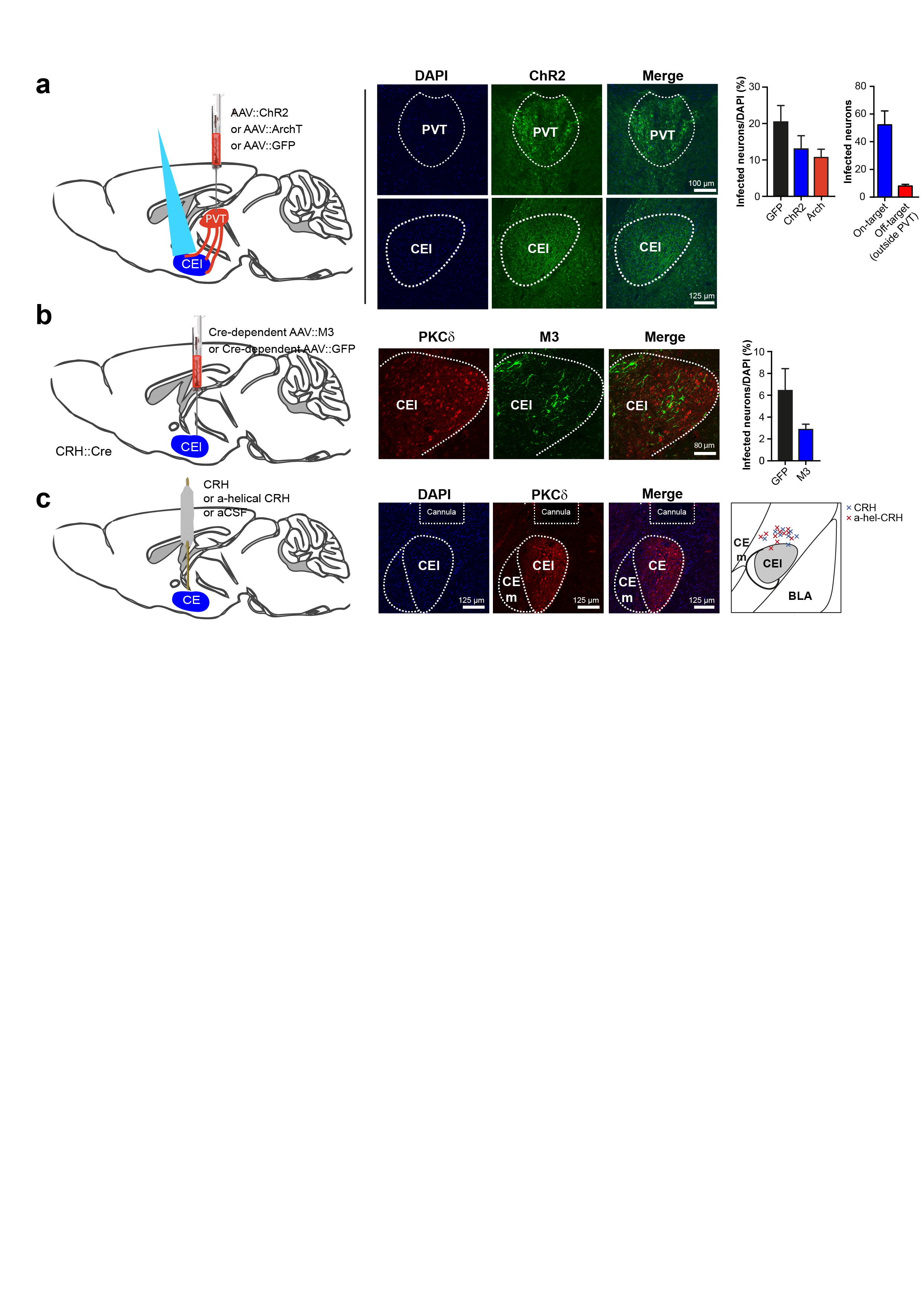


Supplementary Figure S4. Manipulation of PVT-CE circuitry. a Optogenetic manipulation of PVT-to-CEl projections. ChR2, Arch or GFP virus were injected in PVT and optical fibers were placed above CEl (*left*). Expression was limited to PVT and terminal fields in CEl due to viral expression in PVT (*middle*). Percentage of infected neurons over total nuclei in PVT and the number of on-target (inside PVT) and off-target (outside PVT) infected neurons in representative 20 μm slices (*right*). b Pharmacogenetic manipulation of CEl CRH^+^ neurons in CRH::Cre transgenic animals (*left*), with Cre-dependent AAV5 expressing DREADD-hM3Gq identified by IHC against GFP (PKCδ IHC was used to indicate CEl location) (*middle*) and the percentage of infected neurons over total nuclei in CEl (*right*). c Local pharmacological modulation of CEl CRH signaling (*left*). Cannula placement for intracerebral drug administration above CEl, marked by the presence of PKCδ (*middle*). Cannula positions in the mice included in the analysis (*right*).


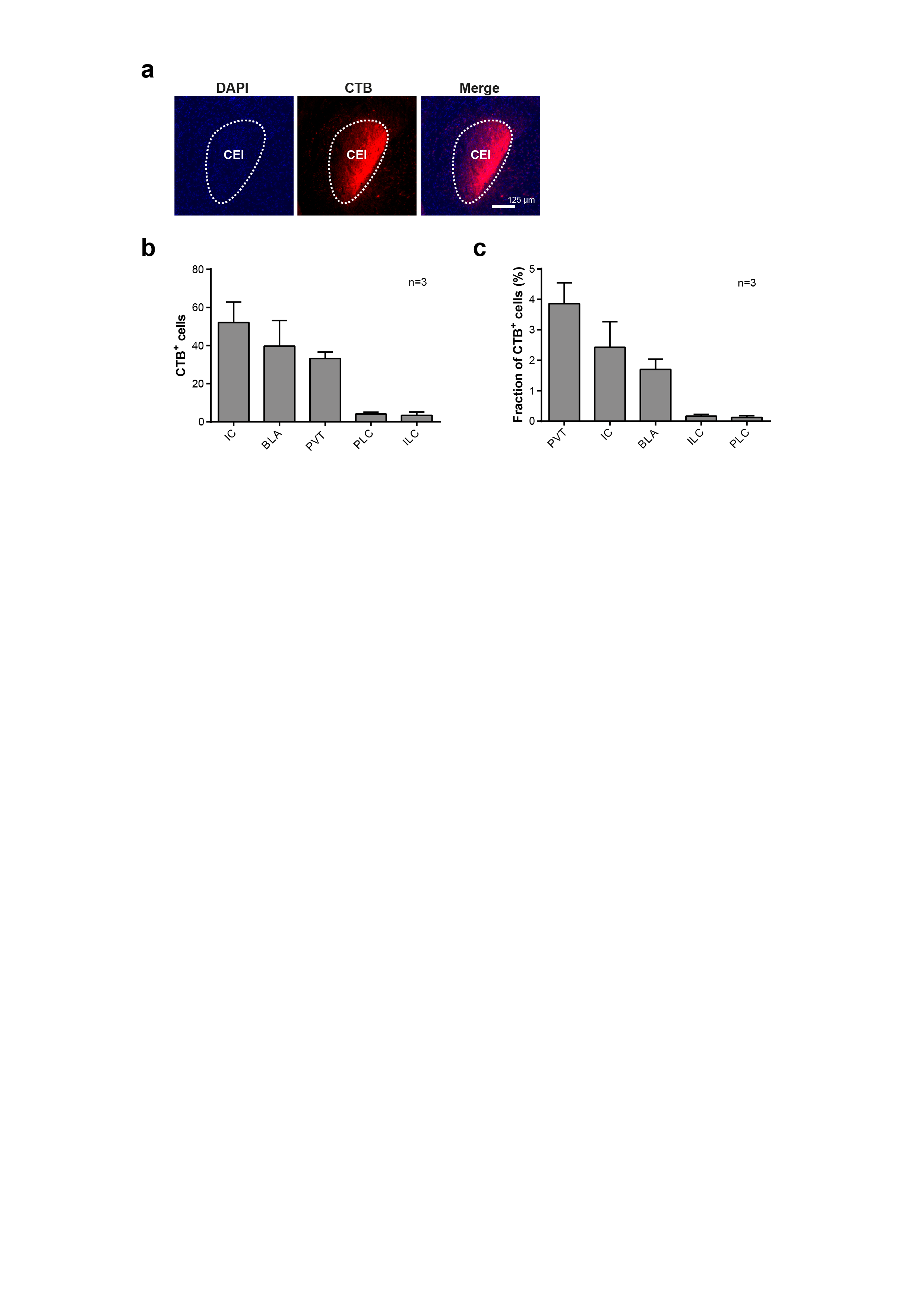


Supplementary Figure S5. The CTB retrograde tracing of projections to CEl. a CTB injection site was limited to CEl. b Identification of most dominant forebrain inputs to CEl by CTB backlabelling cells. CTB positive cells in representative sections from each region. c Estimate of the dominance of CEl projections in CEl projecting forebrain regions, normalized to cell numbers.

IC, insular cortex; BLA, basolateral amygdala, PLC, prelimbic cortex; ILC, infralimbic cortex.


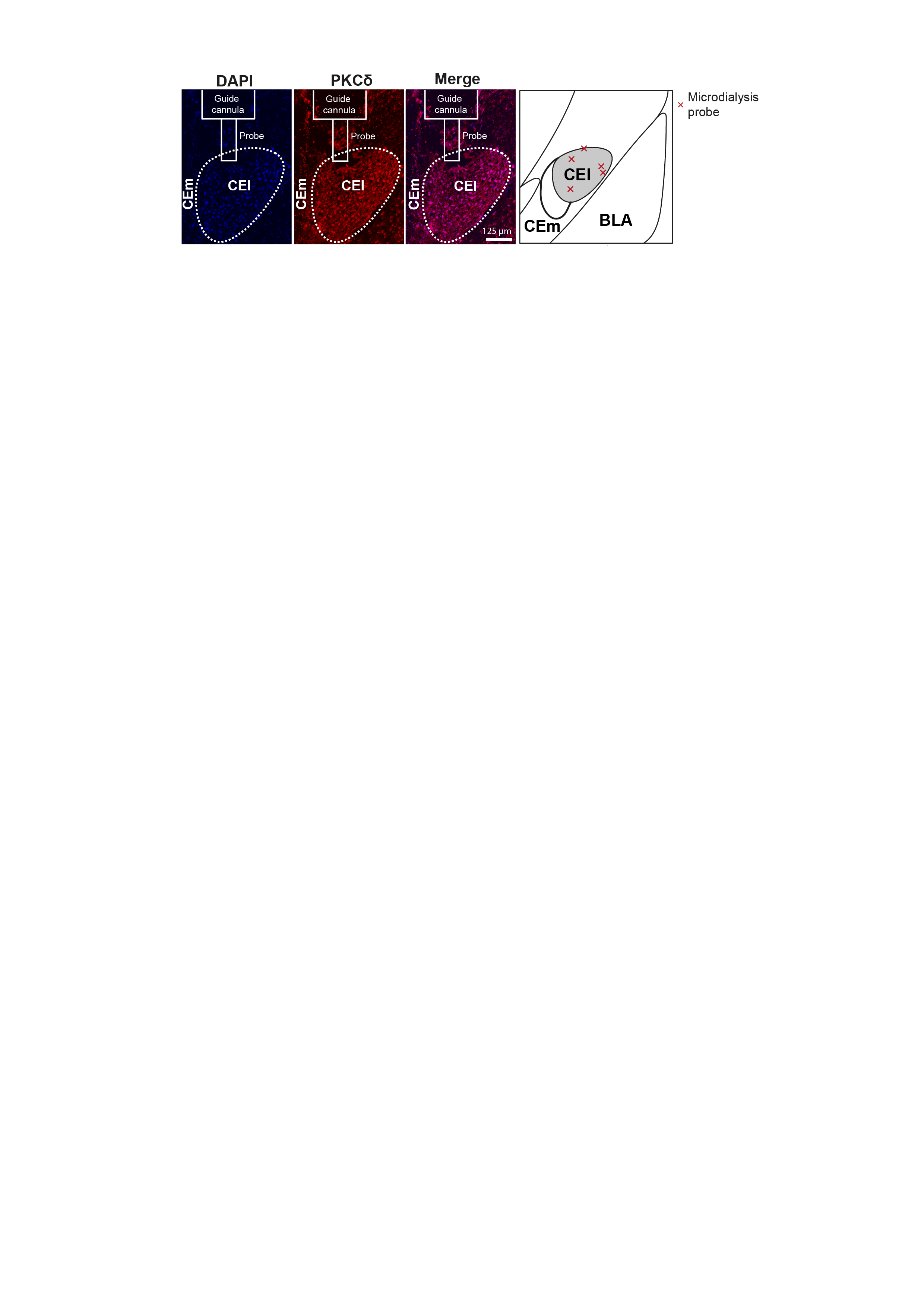


**Supplementary Figure S6**. Cannula placement and probe placement for microdialysis in CE, marked by the presence of PKCδ (*left*). Probe positions of the mice included in the analysis (*right*).


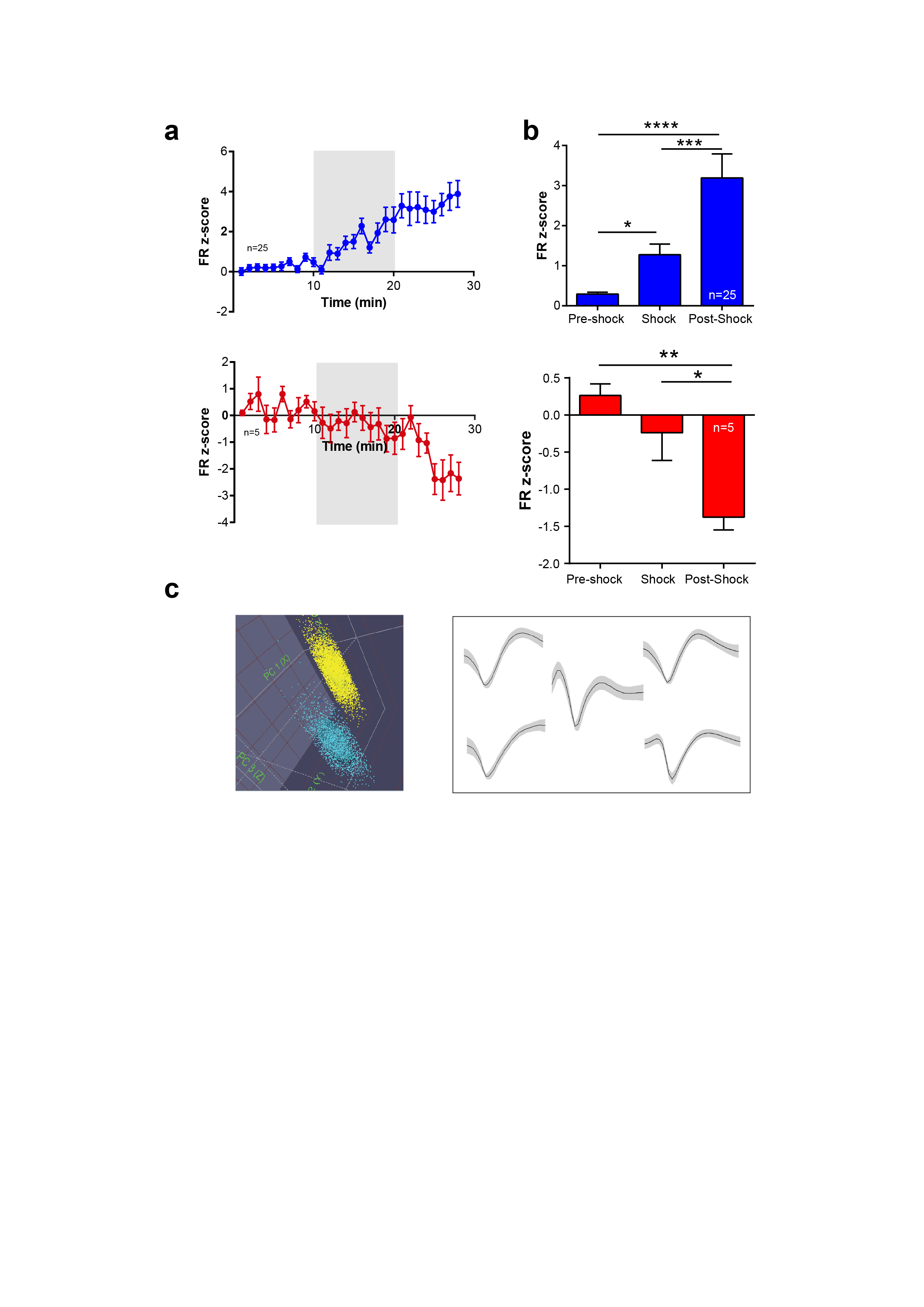


Supplementary Figure S7. Tonic shock responses in PVT during extracellular recordings of lightly anesthetized animals. a *Left,* Time courses (10 min prior, 10 min during shock and 10 min post-shock) of the average firing rate (FR) of isolated units with increased (blue) or decreased (red) firing. The gray bars represent shock. b Comparison of neuronal activity during the pre-, shock, and post-shock periods (RM one-way ANOVA for increased firing p_Time_<0.001, RM one-way ANOVA for decreased firing p_Time_=0.064, Holm-Sidak Post hoc tests) (n indicates neurons extracted from 7 animals). c Cluster separation (*left;* clustering indices J3 = 12.4297, Davies-Bouldin = 0.243337) and example waveforms (*right*) of units from extracellular recordings in PVT. Significance levels are given as * p<0.05, ** p<0.01, *** p<0.001, **** p<0.0001.


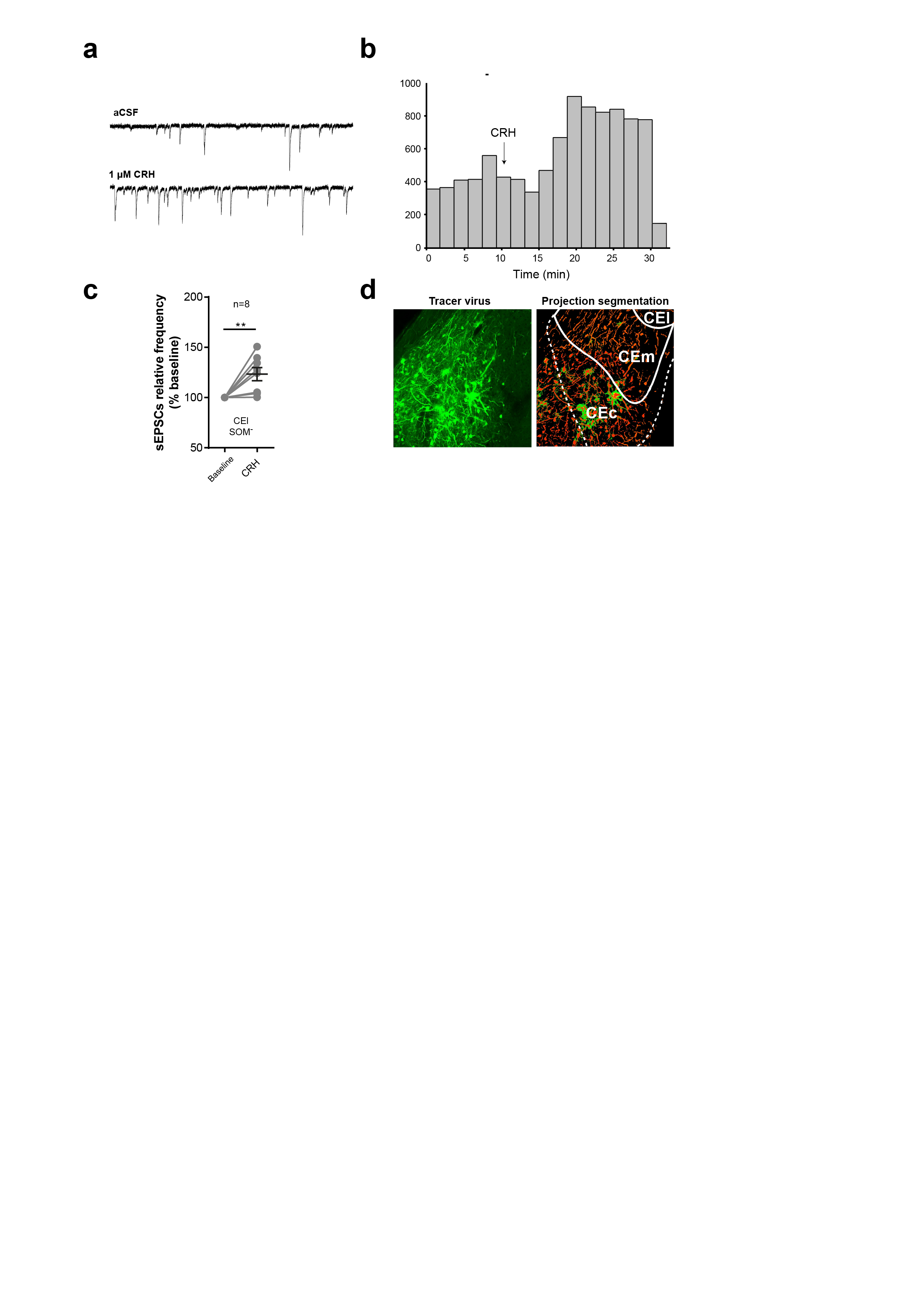


Supplementary Figure S8. CRH signalling in CE. a Representative traces before and after 1 μΜ CRH application. b A neuron responding to bath application of CRH with increased EPSCs. c sEPSCs in CEl SOM^-^ neurons, which correspond largely to CEl PKCδ^+^ cells. Averages during 4 min baseline and during 4 min bath application of CRH (paired t-test p=0.0030). d CRH^+^ cells in the lateral/capsular CE project to CEm. Axonal projections were traced after Cre-dependent AAV::GFP injection into the CE of CRH::Cre animals. Data taken from <http://www.brain-map.org/> (Experiment 267152406 – CEA), with modifications. Significance levels are given as * p<0.05, ** p<0.01, *** p<0.001, **** p<0.0001.


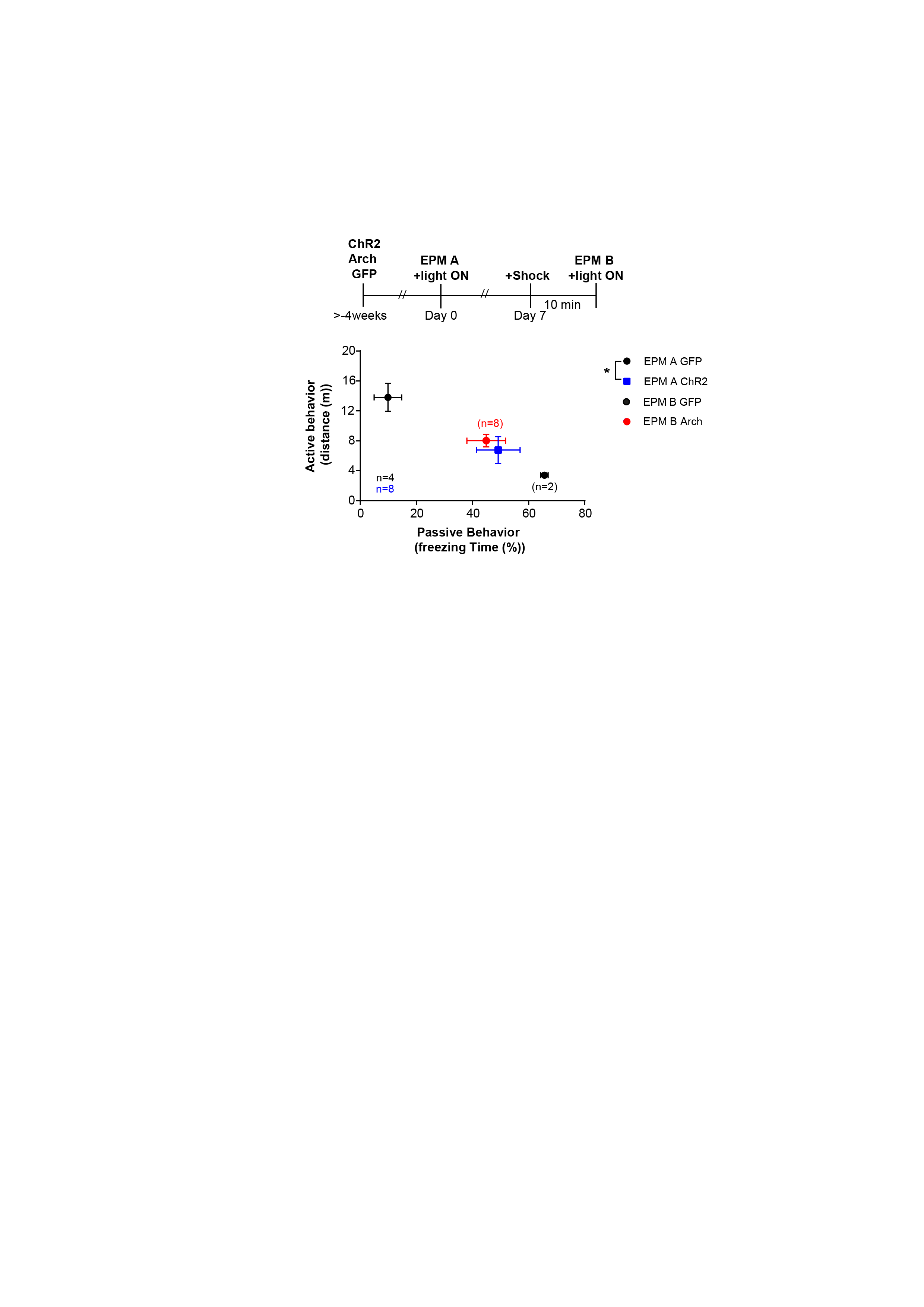


Supplementary Figure S9. Optogenetic manipulation of BLA-to-CEm projections in EPM A or EPM B. BLA-to-CEm activation by ChR2 increased passive responding in EPM A (MANOVA p=0.03, Hotelling T^2^). Note that the BLA-to-CEm inactivation is statistically underpowered and only shown for illustrative purposes. Inactivation in EPM A did not show a significant effect (data not sown).

Significance levels are given as * p<0.05, ** p<0.01, *** p<0.001, **** p<0.0001.


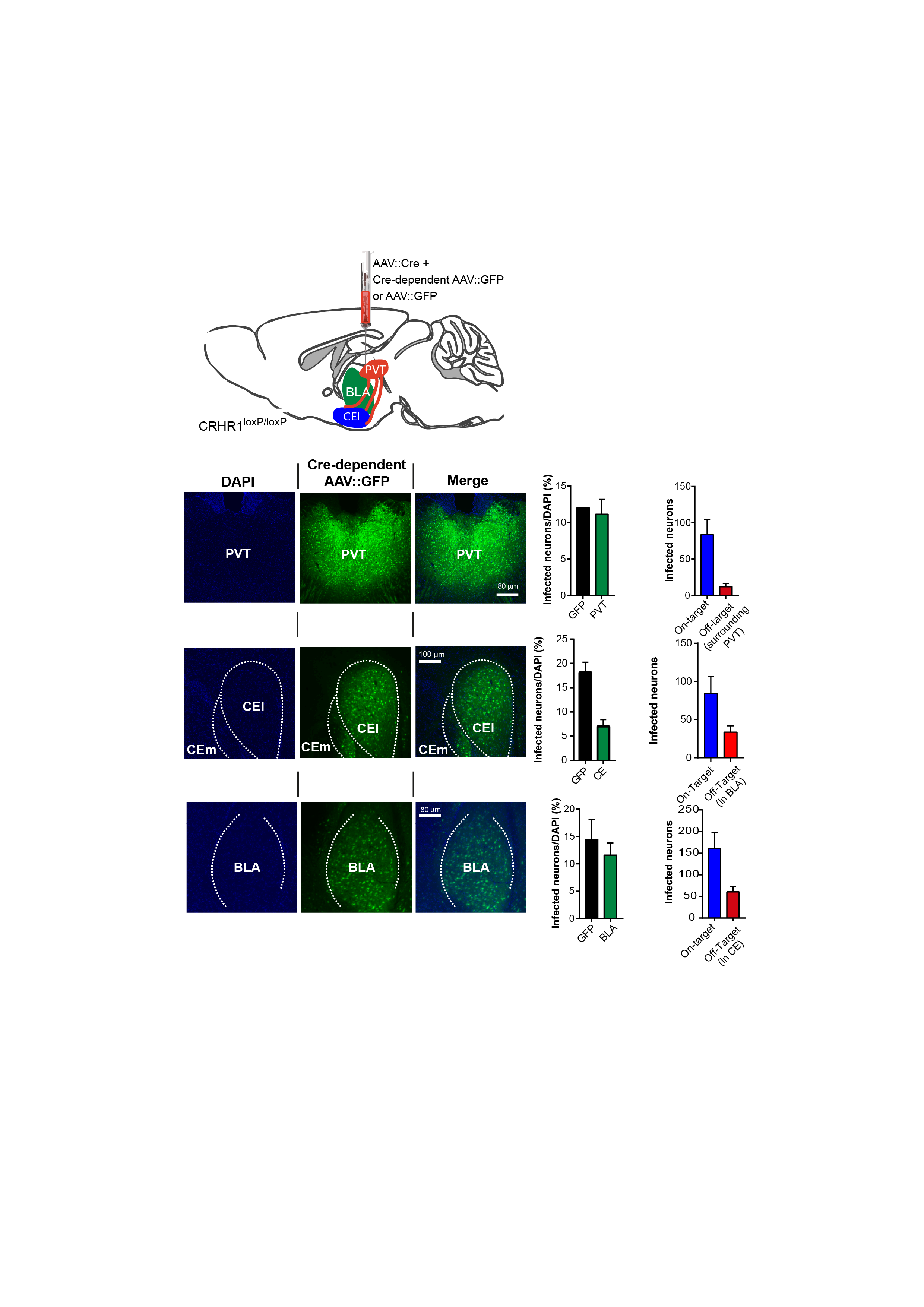


**Supplementary Figure S10.** Region specific knock out of CRHR1. AAV::Cre and AAV::GFP were injected in CRHR1^loxP/loxP^ homozygous mice into PVT (*top*), CEl (*middle*) or BLA (*bottom*). The percentage of infected neurons over total nuclei in PVT, CEl and BLA and the number of on-target and off-target infected neurons in representative 20 μm slices.

**
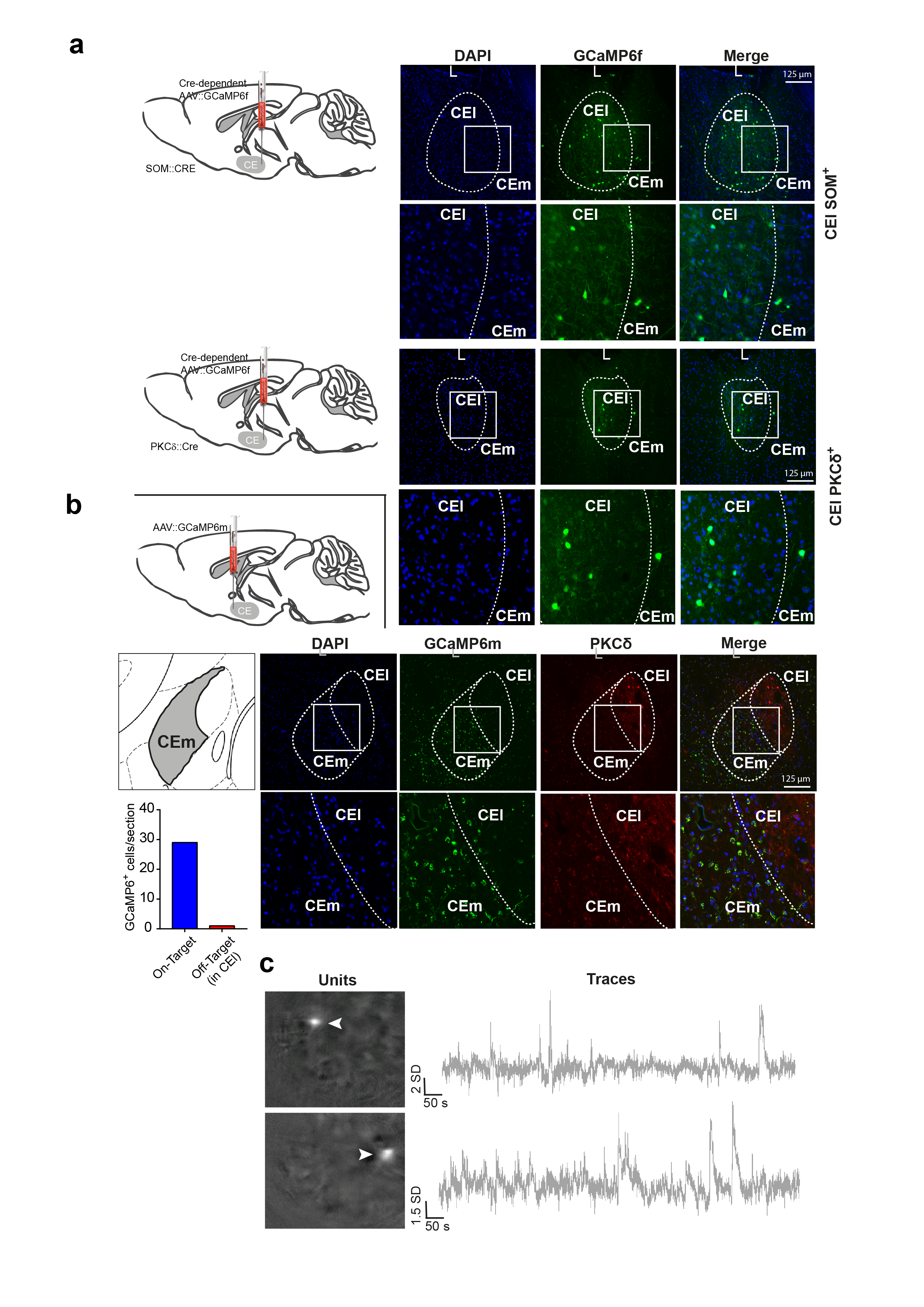
**

**Supplementary Figure S11.** Calcium imaging of CE neurons. **a** Stereotactic injection of Cre-dependent GCaMP6f to target PKCδ^+^ and SOM^+^ neurons and expression of GCaMP6f in CEl. PKCδ staining is used to indicate the location of CEl. **b** *Left,* Stereotactic injection of GCaMP6m to target CEm output neurons and expression of GCaMP6m in CE. *Right*, Quantification of the selectivity of the GCaMP6m expression. Off-target is defined as CEl. **c** Example isolated units for CE recordings. Isolated units from putative CEm neurons (arrowheads) and corresponding Ca^2+^ traces of these units in behaving animals. L indicates approximate position of the imaging lens.

**
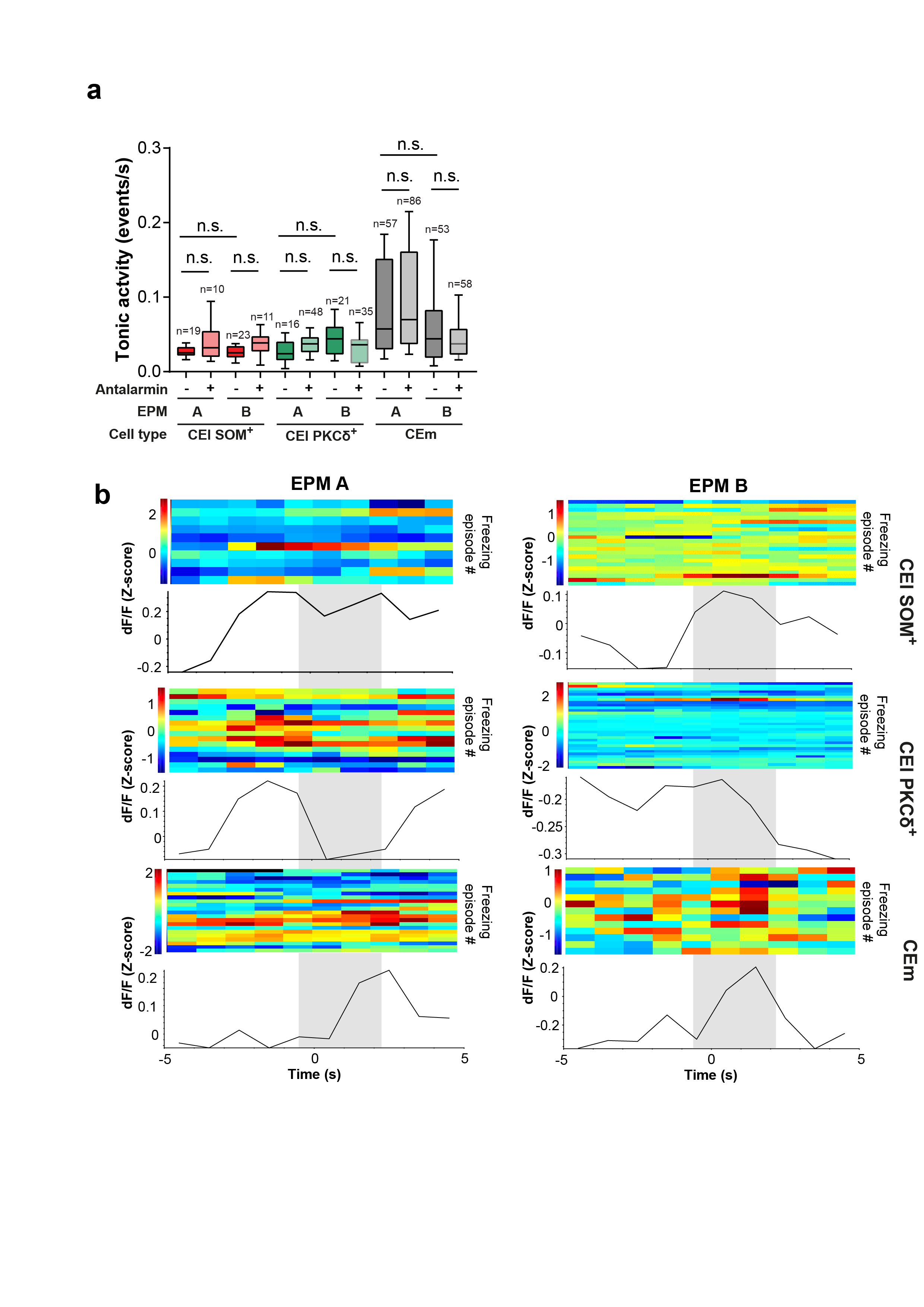
**

**Supplementary Fig. S12.** Modulation of neuronal activity during behavioral challenge by stress experience and CRH antagonists. **a** Tonic activity (Ca^2+^ events/s) of CE neurons during behavioral challenge. No significant differences could be detected. (Kruskal-Wallis test p<0.0001, Dunn’s post hoc test) (n indicates neurons extracted from 1-4 animals). **b** Peri-freezing histograms of Ca^2+^ signals of SOM^+^ and PKCδ^+^ CEl units and putative CEm units with phasic freezing responses. *Top*, peri-event activity during single freezing episodes, *bottom*, peri-event average.

**
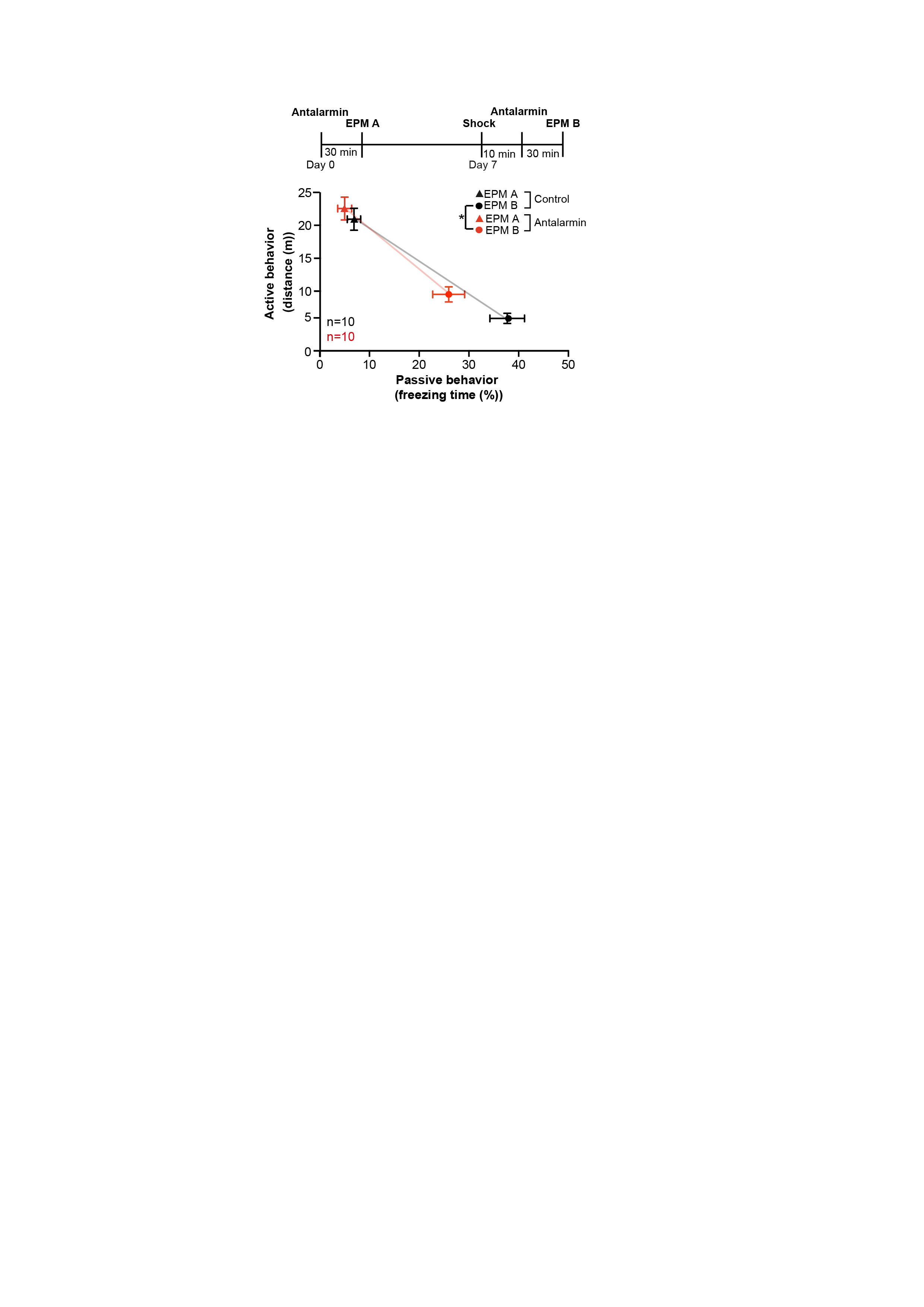
**

**Supplementary Figure S13.** Systemic CRHR1 antagonist antalarmin reverts stress related behavioral plasticity. *Top*, antalarmin was injected systemically before both EPM exposures. *Bottom*, antalarmin injected prior EPM B reduces passive behaviors (MANOVA p<0.0001). In this experiment, the time between shock context exposure and EPM B was 30 minutes to allow time for the drug to act. Significance levels are given as * p<0.05, ** p<0.01, *** p<0.001, **** p<0.0001.

**
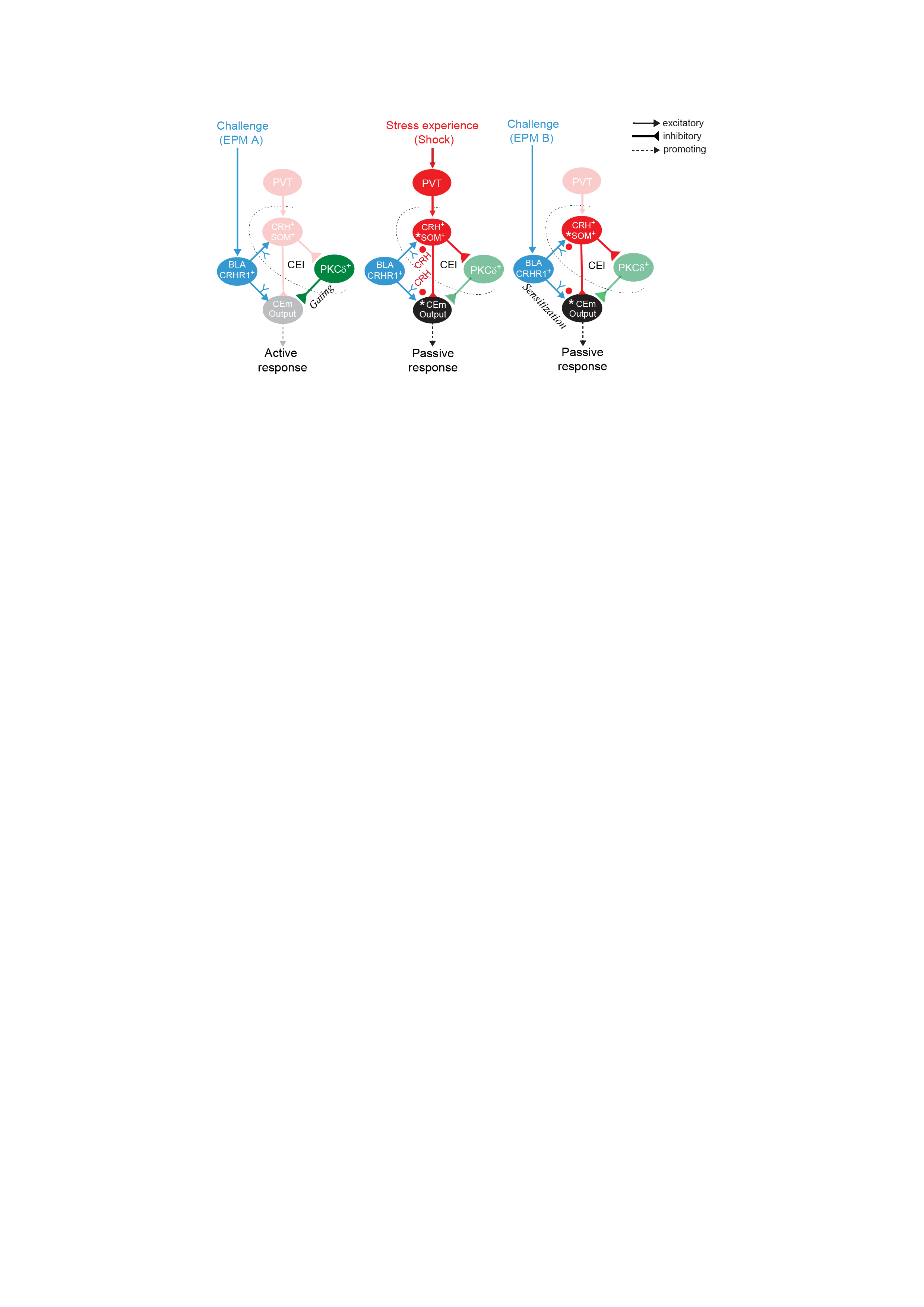
**

**Supplementary Figure S14.** Proposed mechanism for stress and CRH control of passive behavioral responding. CRH^+^/SOM^+^ cells have been combined as they are similar with respect to their behavioral function in fear ^7^ which is distinct from PKCδ^+^ neurons ^8,9^. BLA CRHR1 (Y) facilitates BLA-CE signalling in response to stress, similar to CE CRHR1 (*) in Pavlovian fear ^7^. See discussion for details.
